# Surface molecules of extracellular vesicles secreted by the helminth pathogen *Fasciola hepatica* direct their internalisation by host cells

**DOI:** 10.1371/journal.pntd.0007087

**Published:** 2019-01-18

**Authors:** Eduardo de la Torre-Escudero, Jared Q. Gerlach, Adam P. S. Bennett, Krystyna Cwiklinski, Heather L. Jewhurst, Kathryn M. Huson, Lokesh Joshi, Michelle Kilcoyne, Sandra O’Neill, John P. Dalton, Mark W. Robinson

**Affiliations:** 1 Institute for Global Food Security, School of Biological Sciences, Queen’s University Belfast, Belfast, Northern Ireland, United Kingdom; 2 Glycoscience Group, Advanced Glycoscience Research Cluster, National Centre for Biomedical Engineering Science, National University of Ireland Galway, Ireland; 3 Carbohydrate Signalling Group, Discipline of Microbiology, School of Natural Sciences, National University of Ireland Galway, Ireland; 4 School of Biotechnology, Dublin City University, Dublin 9, Republic of Ireland; University of Melbourne, AUSTRALIA

## Abstract

Helminth parasites secrete extracellular vesicles (EVs) that can be internalised by host immune cells resulting in modulation of host immunity. While the molecular cargo of EVs have been characterised in many parasites, little is known about the surface-exposed molecules that participate in ligand-receptor interactions with the host cell surface to initiate vesicle docking and subsequent internalisation. Using a membrane-impermeable biotin reagent to capture proteins displayed on the outer membrane surface of two EV sub-populations (termed 15k and 120k EVs) released by adult *F*. *hepatica*, we describe 380 surface proteins including an array of virulence factors, membrane transport proteins and molecules involved in EV biogenesis/trafficking. Proteomics and immunohistochemical analysis show that the 120k EVs have an endosomal origin and may be released from the parasite via the protonephridial (excretory) system whilst the larger 15k EVs are released from the gastrodermal epithelial cells that line the fluke gut. A parallel lectin microarray strategy was used to profile the topology of major surface oligosaccharides of intact fluorogenically-labelled EVs as they would be displayed to the host. Lectin profiles corresponding to glycoconjugates exposed on the surface of the 15 K and 120K EV sub-populations are practically identical but are distinct from those of the parasite surface tegument, although all are predominated by high mannose sugars. We found that while the *F*. *hepatica* EVs were resistant to *exo*- and *endo*-glycosidases, the glyco-amidase PNGase F drastically remodelled the surface oligosaccharides and blocked the uptake of EVs by host macrophages. In contrast, pre-treatment with antibodies obtained from infected hosts, or purified antibodies raised against the extracellular domains of specific EV surface proteins (DM9-containing protein, CD63 receptor and myoferlin), significantly enhanced their cellular internalisation. This work highlights the diversity of EV biogenesis and trafficking pathways used by *F*. *hepatica* and sheds light on the molecular interaction between parasite EVs and host cells.

## Author summary

Over the last decade, it has become recognised that extracellular vesicles (EVs) are important mediators of communication by transferring molecular signals (including proteins, lipids, complex carbohydrates, mRNA, microRNA and other non-coding RNA species), between cells. Variously described as exosomes or microvesicles depending on their cellular origin and mode of biogenesis, EVs perform a variety of roles in the maintenance of normal physiology but also participate in pathological settings. EVs also play an important role in host-pathogen interactions, with recent work suggesting that they contribute to helminth immunomodulatory strategies. Here we have identified the proteins and sugars displayed on the outer surface of two sub-types of EVs released by the helminth pathogen *Fasciola hepatica*. We show that the proteins are antigenic and direct EV internalisation by host macrophages. Our study provides a better understanding of how parasite-derived EVs interact with host cells which is important for future development of therapeutics/vaccines that target this interface.

## Introduction

Helminth infections have a major impact on human and animal health in many parts of the world. Indeed, up to one third of the global human population [1] and >50% of the world’s livestock play host to helminth parasites [2]. Helminths cause chronic infections and their persistence within their mammalian hosts is due, in part, to their striking ability to avoid/modulate the host immune response. We, and others, have previously shown that the liver fluke *Fasciola hepatica* secretes several immunomodulatory molecules with multiple mechanisms of action. These include peroxiredoxin that stimulates the development of M2 macrophages [3,4], cathepsin L1 that degrades toll-like receptor 3 within the endosome of macrophages [5] and helminth defence molecule (HDM) that impairs antigen processing and presentation by macrophages by inhibition of vATPase [6]. Secretion of these soluble proteins and shedding of glycoproteins/proteins expressed on the tegumental surface [7,8] was previously thought to be the sole route of export for *F*. *hepatica* antigens. However, it is now recognised that the parasite also releases extracellular vesicles (EVs) as a mechanism for non-canonical secretion of proteins and other molecules [9,10].

Parasite-derived EVs can be internalised by host immune cells resulting in a range of intracellular signalling events that have potent immunomodulatory effects [11]. The major advantage for the parasite with such a mechanism of export and delivery is the ability to package a large array of (perhaps complementary) effector molecules in a protective environment. Thus, protein and RNA molecules that would otherwise be susceptible to degradative enzymes in the host extracellular microenvironment are protected and can exert their biological effects at sites distant from their site of release.

Little is known about the mechanism of uptake of EVs by host cells and whether the process is mediated by specific molecular interactions or by more passive means. One explanation for this is the lack of knowledge pertaining to the molecules specifically displayed on the surface of parasite-derived EVs that could mediate uptake by host cells via initial interaction with plasma membrane receptors. Studies using EVs, derived from various mammalian cell types, have shown that their internalisation by recipient cells can be influenced by both surface-exposed proteins and their post-translational modifications, notably glycosylation [12]. To fully understand the transfer of EV-packaged parasite molecules to host cells, which would facilitate the design of therapeutic blocking agents, it is critical that we identify the cohort of proteins that are exposed on the outer membrane surface of parasite EVs and the displayed EV surface glycosylation.

We have previously shown that *F*. *hepatica* releases at least two EV sub-populations (termed 15k and 120k EVs) that differ according to size and cargo molecules [10,13]. In this study, we used a membrane-impermeable biotin reagent to label the EV surface and then mass spectrometry to identify the labelled proteins of both EV sub-populations. Fluorescently labelled EVs were profiled by multiplexed lectin microarrays to examine the topology of the major carbohydrate motifs presented to the host. Our proteomics and immunohistochemical studies indicate that the 15k EVs originate within the parasite gut whereas the 120k EVs may be released from the excretory system. We also demonstrate that *F*. *hepatica* EV proteins are recognised by host antibodies, with the strength of the host response dependent on the timing of infection. Pre-treatment of both 15k and 120k EVs with host antisera enhanced their uptake by macrophages, an effect that could be replicated using antibodies raised against specific EV surface proteins including DM9 protein, myoferlin and CD63 receptor. The results represent the most definitive characterisation of the surface architecture of EVs from any parasite species to date, and provides a template for a greater understanding of the interactive mechanism(s) between parasite-derived EVs and recipient host cells.

## Methods

### Isolation of adult F. hepatica extracellular vesicles

Adult *F*. *hepatica* parasites were obtained from sheep livers from local abattoirs. To prepare secretions, adult flukes were thoroughly washed with PBS to void their gut contents and to remove any host contaminants and then maintained in RPMI-1640 culture medium containing 0.1% glucose, 100 U penicillin and 100 μg/ml streptomycin (Sigma-Aldrich), at 1 worm/ml (typically in groups of 50 parasites) for 5 h at 37°C. Whilst other methods, such as gradient centrifugation, are increasingly used for EV isolation, we specifically used the differential centrifugation protocol described by Marcilla et al. [9] to specifically characterise the *F*. *hepatica* 15k and 120k EVs we previously isolated using this method [10]. Briefly, after the incubation period, the parasite culture media was collected and centrifuged at 300 x *g* for 10 min and then at 2000 x *g* for 30 min to remove eggs, cells and large debris. The resulting supernatant was centrifuged at 15,000 x *g* for 45 min at 4°C to obtain large vesicles (15k EVs). Culture supernatants were then filtered using a 0.2 μm ultrafiltration membrane, and centrifuged at 120,000 x *g* for 1 h at 4°C to recover smaller vesicles (120k EVs). Both, 15k and 120k EVs were subsequently washed with PBS and immediately used for surface biotin labelling or snap frozen and stored at -80°C for further experiments.

### Surface biotinylation of F. hepatica EVs and streptavidin pulldown

Freshly collected EVs were immediately incubated with a solution containing 1 mg/ml of sulfo-NHS-LC-biotin (ThermoFisher Scientific) in PBS (pH 7.4) for 30 min at 4°C. To prevent hydrolysis of the sulfo-NHS-LC-biotin, the reagent was prepared immediately before use. After labelling, excess biotinylation reagent was neutralized by incubating EVs with 50 mM Tris–HCl in PBS for 15 min at 4°C. Biotinylated samples were subsequently centrifuged either at 15,000 x *g* for 45 minutes or 120,000 x *g*/1 h at 4°C to remove the quenching solution. Labelled EVs were then resuspended in PBS and stored at -80°C until use. The same procedure, excluding the biotin reagent, was followed to prepare non-biotinylated control EVs. Proteins of biotinylated and non-biotinylated EVs were sequentially extracted in three steps with 200 μl of lysis buffer (0.1% SDS, 1% Triton X-100, in 50 mM Tris, pH 7.4) containing CompleteMini protease inhibitor cocktail (Roche Diagnostics). For each extraction, samples were sonicated three times for 10 sec in a water bath sonicator. Samples were then incubated on ice for 30 min with brief vortexing every 5 min and finally centrifuged at 20,500 x *g* for 30 min at 4°C. Solubilized proteins were pooled and extracts of biotinylated and non-biotinylated EVs were incubated separately with streptavidin–sepharose beads (GE Healthcare) for 1 h at room temperature. The beads were washed four times with 400 μl of 50mM NH_4_HCO_3_, pH 8.0 and proteins digested with 5 ng/μl sequencing grade trypsin (Promega) overnight at 37°C. Finally, beads were transferred to a filter device (Millipore) and peptides recovered by centrifugation at 5,000 x *g* for 1 min.

### Mass spectrometry analysis of biotinylated proteins

Three biological replicates of the *F*. *hepatica* 15k and 120k EVs were biotinylated and analysed by LC-MS/MS. Non-biotinylated negative controls were also analysed (in triplicate) for each sample. Tryptic peptides were dried in a vacuum centrifuge and reconstituted with 10 μl of 0.1% TFA before analysis by LC-MS/MS. Five μl of the resulting suspension were delivered to an analytical column (Eksigen C18-CL NanoLC Column, 3 μm; 75 μm x 15 cm) equilibrated in 5% acetonitrile/0.1% formic acid (FA). Elution was carried out with a linear gradient of 5–35% buffer B in buffer A for 30 min (buffer A: 0.1% FA; buffer B: acetonitrile, 0.1% FA) at a flow rate of 300 nl/min. Peptides were analysed in a nanoESI QqTOF mass spectrometer (5600 TripleTOF, ABSCIEX) operating in information-dependent acquisition mode, in which a 0.25-s TOF MS scan from 350–1250 m/z, was performed, followed by 0.05-s product ion scans from 100–1500 m/z on the 50 most intense 2–5 charged ions. Peak list files were generated by Protein Pilot v4.5 (Applied Biosystems) using default parameters and exported to Mascot v2.4.1 (Matrix Science) for database searching.

### Database searching

All MS/MS samples were analysed using Mascot v2.4.1 (Matrix Science). Mascot was set up to search a database comprised of the gene models identified within the *F*. *hepatica* genome (version 1.0, 101,780 entries; accession PRJEB6687 [14]) assuming trypsin digestion with 1 missed cleavage permitted. Mascot was searched with a fragment ion mass tolerance of 0.60 Da and a parent ion tolerance of 10.0 ppm. Carbamidomethylation of cysteine was specified in Mascot as a fixed modification. Gln->pyro-Glu of the *N*-terminus, oxidation of methionine, dioxidation of methionine, acetyl of the *N*-terminus, BHAc of lysine and NHS-LC-Biotin of the *N*-terminus were specified in Mascot as variable modifications. An additional search against the NCBI database or *Ovis aries* gene models (http://www.ensembl.org/Ovis_aries/Info/Index) was run to identify potential host proteins.

### Criteria for protein identification

Scaffold (version Scaffold_4.7.5, Proteome Software Inc., Portland, OR) was used to validate MS/MS based peptide and protein identifications. Peptide identifications were accepted if they could be established at greater than 95.0% probability by the Scaffold Local FDR algorithm. Protein identifications were accepted if they could be established at greater than 99.0% probability and contained at least 2 identified peptides. Protein probabilities were assigned by the Protein Prophet algorithm [15]. Proteins that contained similar peptides and could not be differentiated based on MS/MS analysis alone were grouped to satisfy the principles of parsimony. Proteins sharing significant peptide evidence were grouped into clusters.

### Filtering of potential affinity chromatography contaminants and construction of the final dataset

The following criteria were used to filter the MS/MS results. Firstly, only proteins with two or more unique peptides reported in at least two of the three replicates were considered for further analysis. In order to remove background contaminants subsequent to affinity chromatography, proteins identified for each EV population were submitted to the CRAPome repository [16] by selecting all available streptavidin-agarose experiments and our non-biotinylated EVs as controls. Significance analysis of interactome (SAINT) score [17] was employed to set the threshold (at 0.66). Additionally, a quantitative analysis was performed in Scaffold using a t-test (Benjamini-Hochberg correction; significance level, p<0.05) as statistical method and emPAI as quantitative method. Normalization was performed with zero as minimum value. Those proteins below the SAINT threshold and/or reported as enriched in the non-biotinylated EVs by Scaffold’s quantitative profile were removed as potential contaminants present due to non-specific interaction with the streptavidin-agarose.

### Immunoblot analysis of F. hepatica EVs

Antibodies to specific *F*. *hepatica* EV surface molecules were prepared in rabbits against peptides derived from the external domains of tetraspanin-CD63 receptor (CD63rec; GAKFTHKDSAAGRT), DM9 domain-containing protein (DM9; MYRHQQPAREVS), myoferlin (MYO; GEPKRAPENIQLRD), PDCD6IP (Alix; TSSKKRPAPDRPPP), acid sphingomyelinase (aSMase; IEKGNDEGYAENKP), TSG101 (KSYKYARDVTNDVK) and Ral-A (NKIDLTQERTVPFE) (GenScript, NY, USA). The protein content of the fluke EVs was determined using the BCA protein assay kit (Pierce). Equal amounts (10 μg) of each sample were run on reducing NuPAGE Novex 4–12% Bis-Tris gels (Life Technologies), and transferred to nitrocellulose membranes (GE Healthcare) at 120 mA for 45 min. Following transfer, the membranes were incubated in blocking solution (TBST1: 20 mM Tris–HCl, 150 mM NaCl, 1% Tween 20, pH 7.6) containing 5% skimmed milk for 2 h at room temperature (18–21°C). Blots were probed with 0.5–1.0 μg/ml of the peptide antibodies or a 1:100 dilution of rat-antiserum taken at time-points (0, 7, 21 and 70 days) post-infection with *F*. *hepatica* for 2 h at room temperature. After washing in TBST1 (3×10 min), an appropriate alkaline phosphatase-conjugated IgG secondary antibody was applied to the membranes for 1 h at room temperature before detection using the BCIP/NBT substrate (Sigma-Aldrich).

### Immunofluorescence microscopy

Adult *F*. *hepatica* were fixed with 4% PFA in 0.1 M PBS (Sigma-Aldrich) overnight at 4°C and subsequently embedded in JB-4 resin (Sigma-Aldrich). Semi-thin sections, 2 μm thick, were cut on a pyramitome and mounted on clean glass slides. For immunofluorescence, JB-4 sections were washed with PBS and then incubated in 10 μg/ml of anti-FhCD63rec or anti-FhRAL-A in antibody diluent (AbD: PBS containing 0.2% (v/v) Triton X-100) or a 1 in 500 dilution in AbD of rabbit anti-serum raised against recombinant *F*. *hepatica* cathepsin L1 overnight at 4°C. As a negative control, comparable sections were incubated in rabbit pre-immune serum. The sections were then washed three times in AbD before incubation in a 1 in 100 dilution of the secondary antibody, fluorescein isothiocyanate (FITC)-conjugated goat anti-rabbit IgG (Sigma-Aldrich), in AbD for 1 h at room temperature. Following three washes in PBS, the sections were mounted in glycerol containing 10% (v/v) PBS and 0.1 M propyl gallate (Sigma-Aldrich) then viewed under a Leica DM2500 fluorescent microscope. Leica type N immersion oil was used in viewing and all images taken at room temperature.

### Fluorescent labelling of FhTeg protein and EVs

The tegumental surface of adult *F*. *hepatica* (FhTeg) was extracted and labelled with Alexa Fluor NHS succinimidyl ester 555 (ThermoFisher Scientific) as previously described [18,19]. *F*. *hepatica* 15k and 120k EVs were fluorescently labelled with the lipophilic dye PKH26 (Sigma-Aldrich) as previously reported [20]. Following labelling, excess dye was removed from EVs using a 100 kDa MWCO spin device (EMD-Millipore).

### Enzymatic modification of F. hepatica EV surface oligosaccharides

Five to fifty units (determined empirically for each enzyme) of *exo*-glycosidase (Jack bean α-mannosidase, Sigma-Aldrich; β-galactosidase from bovine testes, Sigma-Aldrich), *endo*-glycosidase (*endo*-β-*N*-acetylglucosaminidases Endo Tv [21] and Endo H, Roche), and glyco-amidase (*N-*glycoamidase F, PNGase F) were used to modify the surface glycosylation of the EVs. Each enzyme was added to 1 μg of PKH26-labelled EVs (total protein content) in 200 mM sodium phosphate buffer including protease inhibitors (Roche) at pH 4.5 for α-mannosidase and β-galactosidase, pH 5.5 for endoglycosidases and pH 7.4 for glyco-amidase treatment. The reactions, and enzyme-free negative controls, were incubated in the dark for 14 h at 37°C. In parallel, the activity of β-galactosidase and PNGase F were verified by digestion of bovine fetuin under the same conditions used for the EVs. Similarly, the activity of Endo H, Endo Tv and α-mannosidase were verified by digestion with bovine RNAse B.

### Profiling of fluorescently-labeled EVs with lectin microarrays

Lectin microarrays were performed in at least biological duplicate. Titrations with fluorescently labelled vesicles revealed that the optimal concentration range for EV loading was 0.6 to 1.0 μg/mL based on the signal to noise ratio and overall image quality. Following titrations, experiments including multiple technical replicates (n = 3 at minimum) were carried out with PKH26-labeled EVs (0.8 μg/ml total protein concentration). Lectin microarrays were constructed as previously described [20,22]. Briefly, 48 lectins, supplemented with 1 mM of their respective haptenic sugar to maintain binding site integrity were printed on Nexterion H (Schott) functionalized glass slides at 20°C and 62% (+/-2%) relative humidity using a sciFLEXARRAYER S3 non-contact spotter (Scienion, Berlin, Germany). Unoccupied functional groups were rendered inert by exposure to 100 mM ethanolamine in 50 mM sodium borate, pH 8.0, for 1 h. Microarrays were washed in PBS with 0.05% Tween 20 (PBST) three times and once with PBS, centrifuged dry (450 x *g*, 5 min) and stored at 4°C with desiccant until use. During titration, microarray slides were incubated with PKH26-labelled FhEVs (0.08–5.0 μg/ml) diluted in buffer containing 20 mM Tris-HCl, 100 mM NaCl, 1 mM CaCl_2_, 1 mM MgCl_2_ (pH 7.4) with 0.025% Tween 20 (TBST2) for 40 min at 23°C. Slides were then washed in TBST2 for 2 min, rinsed with TBS before drying by centrifugation (450 x *g* for 5 min). The slides were then imaged immediately using the 532 nm (Cy3) channel of an Agilent G2505B microarray scanner. All experiments included a control glycoconjugate in a separate subarray to monitor performance of the arrays and imaging equipment. To confirm that the lectin-EV interactions were carbohydrate mediated, select sugars (lactose (Lac), α-methylmannose (αManOMe), and *N*-acetyl-D-glucosamine (GlcNAc) were also introduced as competitive inhibitors (50 mM final concentration) of EV binding in discrete subarrays.

### Microarray data extraction and analysis

Local background-corrected intensity values were extracted from the image files using GenePix Pro v6.1.0.4 (Molecular Devices) and evaluated in a manner similar to Gerlach et al. [20]. The median of 6 replicate spots per sub-array was handled as a single data point for graphical and statistical analysis. Unsupervised, hierarchical clustering of EV-lectin binding data was performed with Hierarchical Clustering Explorer v3.0 (http://www.cs.umd.edu/hcil/hce/hce3.html). Previously normalized data (all enzyme treatments; total intensity mean adjustment) or raw data scaled inside HCE (FhEV vs FhTeg/ 0 to 30,000 relative fluorescence intensity (RFU)) for EVs was clustered with the following parameters: no pre-filtering, average linkage, Euclidean distance. Statistical significance of lectin binding inhibition by competition with soluble mono- or disaccharides was established by unpaired, two-tailed Student’s t-tests (with unequal variance) of individual lectin data sets in Excel (Microsoft).

### Macrophage uptake assays

PKH26-dyed EVs were incubated with either serum from *F*. *hepatica*-infected rats (0, 7, 21 and 70 days post-infection) or anti-EV antibodies at 1:100 dilution at 4°C overnight. Unbound antibodies were removed by centrifugation and the EV pellets were re-suspended in PBS. RAW264.7 cells were allowed to attach to μ-Slide 8-well chambered coverslips (Ibidi) overnight and the following day they were transferred to DMEM supplemented with exosome-depleted FBS (Gibco) and 1% L-glutamine. Cells were incubated with 5 μg per well of PKH26-dyed EVs (control) or antibody-coated EVs for 3 hours at 37°C with 5% CO_2_. In some experiments, cells were incubated with EVs in the presence of 2μg/ml cytochalasin D (ThermoFisher Scientific). Cells were washed three times in PBS and then fixed with 4% PFA, washed in PBS, counterstained with DAPI and mounted in glycerol containing 10% (v/v) PBS and 0.1 M propyl gallate (Sigma-Aldrich). Slides were examined using a Leica SP5 confocal microscope (Leica Microsystems) with LAS AF software (Leica). Fluorescence was quantified using ImageJ. At least 8 fields were analysed per experiment and a 1-way ANOVA was performed.

## Results

### The surface-exposed proteome of EVs secreted by adult F. hepatica

To identify the proteins displayed on the outer surface of *F*. *hepatica* EVs, intact vesicles were incubated with a membrane-impermeable biotin reagent, sequentially extracted and the biotinylated proteins were captured by affinity chromatography using streptavidin-agarose beads (S1 Fig). On-column trypsin digestion was performed, and the resulting peptides were analysed by LC–MS/MS against the *F*. *hepatica* gene models [14]. Only proteins with two or more unique peptides in at least two of the three biological replicates were accepted. In parallel, non-biotinylated EVs were subjected to the same procedure and quantitative and statistical analysis with these controls allowed the detection of potential contaminants, which were removed from the biotinylated dataset.

**Fig 1:**
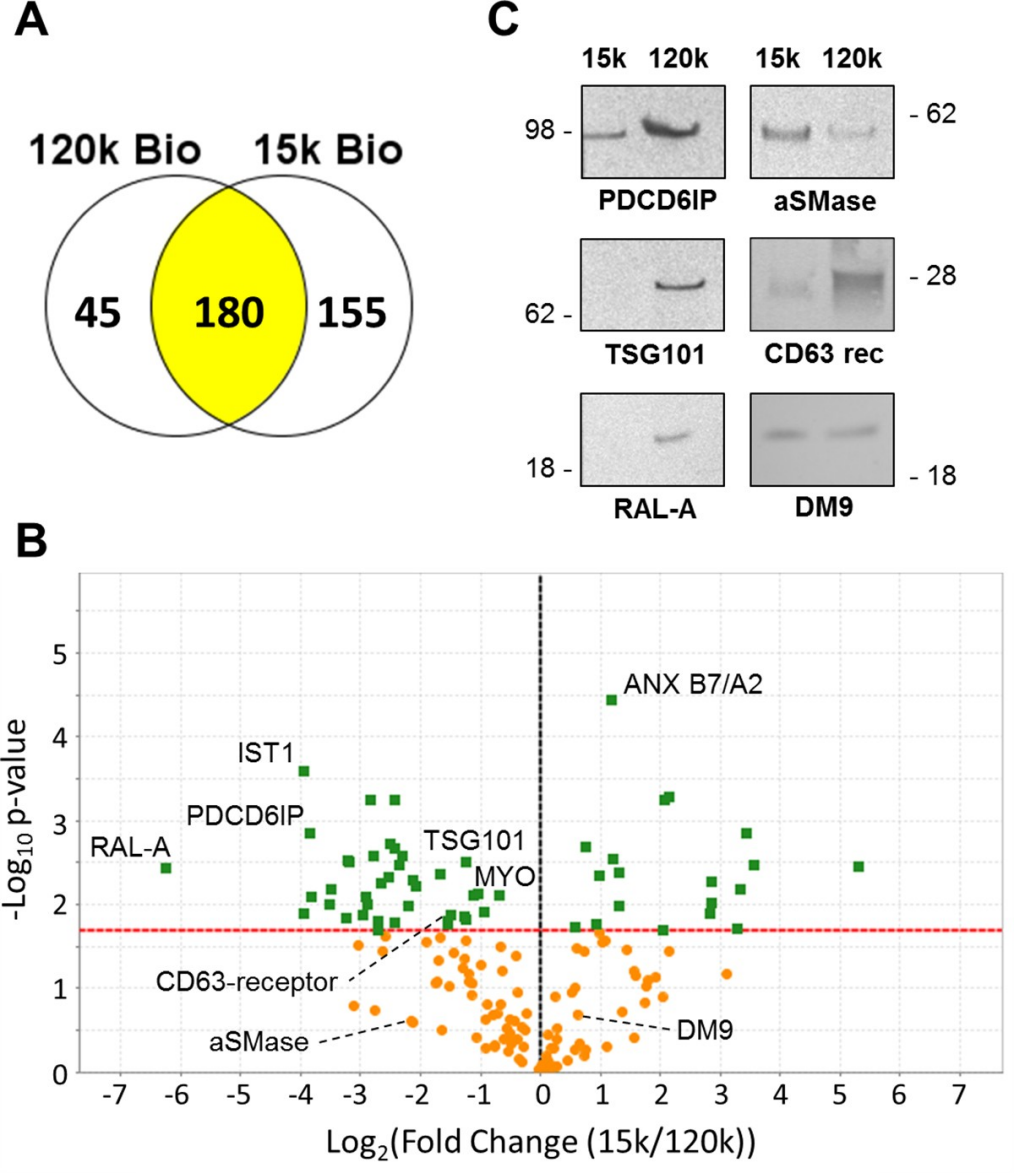
Qualitative and quantitative proteomic analyses of 15k EVs and 120k EVs released by adult *F*. *hepatica* suggest different intracellular origins of the EV sub-populations and identify specific surface proteins. (A) Venn diagram showing the distribution of proteins qualitatively identified in the 15k and 120k EVs (>2 matched peptides in at least 2 out of 3 replicate samples). (B) The 180 surface proteins that were expressed by both the 15k and 120k EVs were subjected to quantitative analysis shown as a Volcano plot. The x-axis represents log_2_(fold-change) (15k/120k EVs) and the y-axis shows the −log_10_ (*p* value). The dashed red line indicates the significance threshold (*p* = 0.05). The vertical dashed black line indicates zero fold change. Green boxes represent proteins whose expression is significantly different between the two EV sub-populations whilst the orange circles represent proteins that did not significantly change. The position of proteins selected for further analysis by Western blot are shown. (C) Equal amounts (10μg total protein) of 15k and 120k EVs were analysed by Western blot using antibodies raised against *F*. *hepatica* proteins identified on the EV surface by LC-MS/MS. The relative expression patterns of the target proteins follows that of the quantitative proteomics analysis.

Using this approach, 380 individual proteins were identified, of which, 180 are shared by 15k and 120k EV populations; 155 proteins were exclusive to the 15k EVs, whereas 45 proteins were present in the 120k EVs only (Fig 1A; S1 Table). Enrichment analysis of Gene Ontology (GO) terms, highlighted exosomes, cytoplasm, lysosomes and plasma membrane as the most abundant terms for both EV populations and subsequent protein domain analysis supported the presence of membrane and membrane-associated proteins (S2 Fig). Indeed, membrane proteins such as pumps, channels and transporters, were amongst the most abundant functional groups identified in the *F*. *hepatica* EVs (Table 1) which validates the biotin labelling and membrane extraction methods used in this study.

**Table 1:** Membrane transport proteins found on the surface of *F*. *hepatica* EVs.

Protein name	Gene ID	*t*-test (*p* value)	emPAI 15k	emPAI 120k
**Pumps**				
ABC subfamily B1 (MDR/TAP)	BN1106_s274B000296	0.0077	0.429	0.934
ABC subfamily D4 (ALD)	BN1106_s3396B000087	0.0021	0.916	4.974
Multidrug resistance protein 1	BN1106_s2471B000098	0.0017	0.383	1.637
Ca^2+^ transporting ATPase	BN1106_s577B000267	0.38	0.045	0.095
Cation-transporting ATPase	BN1106_s181B000525	0.001	0.000	0.151
Cation-transporting ATPase	BN1106_s181B000526	0.0092	0.398	0.000
H+ transporting ATPase	BN1106_s3321B000106	0.01	0.056	0.644
Na^+^/K^+^ transporting ATPase	BN1106_s521B000167	0.0031	0.565	1.342
Phospholipid-transporting ATPase	BN1106_s253B000145	0.24	0.010	0.044
Phospholipid-transporting ATPase	BN1106_s626B000339	0.0033	0.046	0.236
Phospholipid-transporting ATPase	BN1106_s435B000243	0.013	0.103	0.798
Phospholipid-transporting ATPase	BN1106_s435B000242	0.0069	0.000	0.173
Na^+^/K^+^ ATPase	BN1106_s3547B000116	0.73	0.136	0.099
Vacuolar H^+^ ATPase	BN1106_s2593B000233	0.16	0.037	0.317
V-ATPase subunit B	BN1106_s1633B000182	0.037	0.287	0.055
V-ATPase subunit I	BN1106_s18772B000008	0.017	0.759	2.201
V-ATPase subunit I	BN1106_s4862B000066	0.0043	0.099	0.312
V-ATPase subunit C	BN1106_s2545B000160	0.00084	0.086	0.000
V-ATPase subunit H	BN1106_s2350B000136	0.00026	0.208	0.000
**Channels**				
Cation channel M4	BN1106_s1307B000310	0.0014	0.000	0.127
Chloride channel protein 7	BN1106_s2830B000084	0.12	0.000	0.056
**Transporters**				
Anion exchange protein	BN1106_s1377B000259	0.12	0.000	0.064
Anoctamin	BN1106_s1581B000120	0.027	0.187	0.441
Cation/chloride co-transporter	BN1106_s639B000758	0.12	0.065	0.143
Glucose transporter 3	BN1106_s584B000348	0.061	0.088	0.136
Glucose transporter 5	BN1106_s2815B000111	0.18	0.000	0.231
Cationic amino acid transporter 1	BN1106_s2234B000252	0.0019	0.043	0.244
Cationic amino acid transporter 1	BN1106_s1B000532	0.0088	0.263	0.000
Inositol transporter	BN1106_s3611B000052	0.00035	0.000	0.193
Phosphatidylinositol transfer protein	BN1106_s1214B000318	0.0023	0.175	0.000
Permease 1 heavy chain	BN1106_s3721B000192	0.095	0.161	0.047
Phosphatidylcholine transfer protein	BN1106_s538B000493	0.088	0.103	0.225
Putative amino acid permease	BN1106_s1490B000080	0.024	0.207	0.657
Putative sodium/solute symporter	BN1106_s1326B000429	0.083	1.107	2.519
Solute carrier family 2 member 3	BN1106_s584B000350	0.014	0.304	0.813
Solute carrier family 43 member 3	BN1106_s6088B000072	0.086	0.063	0.210

Several cysteine, serine and metallo- peptidases were found on the EV surface. Of the cysteine peptidases, cathepsin Bs were of similar abundance in both EV sub-populations whilst cathepsin L1, L2 and a cathepsin D-like aspartic protease were enriched in the 15k EVs (Table 2). Whilst the metallo-peptidases were generally more abundant on the surface of the 15k EVs, leucine aminopeptidases were found in both vesicle populations [10]. A number of protease inhibitors (serpins, cystatin and a multi-domain cystatin) were also found in the surface-biotinylated proteins of both the 15k and 120k EVs (Table 2). Other dominant functional groups of EV surface proteins included signalling proteins (including a large number of molecules responsive to calcium enriched in the 15k EVs), metabolic enzymes, receptors and carrier proteins (S1 Table).

**Table 2:** Proteases and protease inhibitors found on the surface of *F*. *hepatica* EVs.

Protein name	Gene ID	*t*-test (*p* value)	emPAI 15k	emPAI 120k
**Cysteine peptidases**				
Cathepsin B6/8	BN1106_s793B000177	0.099	1.840	1.231
Cathepsin B4/5/7	BN1106_s13444B000002	0.46	1.099	1.543
Cathepsin B3	BN1106_s1772B000188	0.57	0.124	0.180
Cathepsin L1	BN1106_s10332B000011	0.036	0.082	0.018
Cathepsin D-like aspartic protease	BN1106_s2719B000195	0.0016	0.076	0.000
Cathepsin L2	BN1106_s8098B000020	< 0.00010	0.116	0.000
Calpain	BN1106_s204B000249	0.019	0.844	0.565
Calpain B	BN1106_s368B000158	0.0052	0.170	0.023
Calpain 4/6/7	BN1106_s2697B000090	0.0065	0.393	0.000
Calpain 7	BN1106_s1630B000272	0.0026	0.040	0.199
Legumain-4/5	BN1106_s1861B000097	0.11	0.598	0.773
**Serine peptidases**				
Carboxypeptidase C	BN1106_s1241B000264	0.012	0.410	0.787
Pro-X carboxypeptidase	BN1106_s1620B000120	0.038	0.126	0.345
Acylaminoacyl-peptidase	BN1106_s3345B000120	0.0007	0.068	0.000
Mastin	BN1106_s5880B000098	0.88	0.117	0.101
**Metallo peptidases**				
Leucine aminopeptidase 2	BN1106_s617B000566	0.029	1.803	0.879
Leucine aminopeptidase	BN1106_s5268B000042	0.0033	4.759	0.403
Aminopeptidase P1	BN1106_s997B000230	0.13	0.033	0.000
Xaa-Pro dipeptidase	BN1106_s468B000343	0.002	1.039	0.615
Nardilysin	BN1106_s2211B000138	0.019	0.098	0.000
Dipeptidyl-peptidase III	BN1106_s13034B000002	0.12	0.032	0.000
Thimet oligopeptidase	BN1106_s8350B000044	0.12	0.089	0.000
**Protease inhibitors**				
Serpin 7	BN1106_s3864B000104	0.076	0.347	0.074
Serpin B6	BN1106_s1727B000096	0.41	0.478	0.624
Serpin B	BN1106_s4618B000050	0.066	0.243	0.557
Serpin B6	BN1106_s122B000261	0.5	0.457	0.554
Serpin B	BN1106_s3226B000049	0.49	0.210	0.355
Cys1 protein	BN1106_s1612B000138	0.00011	0.051	0.000
Cystatin-1	BN1106_s4651B000094	0.24	1.448	1.938

MS/MS data was also searched against the *Ovis aries* genome to identify any host proteins associated with the EV surface. No host proteins were associated with the 120k EVs; however, several were identified using the 15k MS/MS data including annexin, ceruloplasmin, HSP70, vitronectin and valosin-containing protein.

### Qualitative and quantitative proteomics analysis suggests different modes of biogenesis of the F. hepatica EV sub-populations

Extensive proteomics analysis was performed on three biological replicates of the biotin-bound surface protein fractions of the *F*. *hepatica* EVs. In addition to the 155 and 45 proteins that were unique to the 15k and 120k EVs respectively, 180 proteins were common to both vesicle sub-populations (Fig 1A). A label-free quantitative approach (based on emPAI values) was used to determine the respective levels of these shared proteins. Volcano plot representation of the relative abundance of EV proteins identified several proteins of the ESCRT pathway, and others involved in vesicle trafficking that were significantly (*p* < 0.05) enriched in the 120k EVs. These included IST1, RAL-A, TSG101, PDCD6IP (Alix) and CD63 receptor. A further three ESCRT proteins (STAM-binding protein, CHMP1A and CHMP2A) were exclusive to the 120k EVs (Table 3). In contrast, proteins involved in plasma membrane remodelling (annexins, phospholipase A2, Na^+^/H^+^ exchange regulatory cofactor NHE-RF2), proteases, cytoskeletal and cytosolic proteins were significantly enriched (*p* < 0.05) in the 15k EVs (Fig 1B; Tables 2 and 3; S1 Table). These results indicate that 120k EVs are derived from the endosomal compartment, whereas 15k EVs originate from the cytoplasm, hence carrying more cytoskeletal/proteases/cytosolic proteins.

**Table 3:** Proteins associated with EV biogenesis and vesicle trafficking found on the surface of *F*. *hepatica* EVs.

Protein name	Gene ID	*t*-test (*p* value)	emPAI 15k	emPAI 120k
**ESCRT components**				
STAM binding protein	BN1106_s2325B000322	0.14	0.000	0.169
TSG101	BN1106_s410B000432	0.0052	0.080	0.352
CHMP2A	BN1106_s912B000169	0.0045	0.000	0.980
CHMP1A	BN1106_s2655B000264	0.047	0.000	1.393
CHMP1B	BN1106_s2316B000077	0.0081	0.111	1.570
IST1	BN1106_s3747B000112	0.00025	0.073	1.136
VPS4B	BN1106_s1437B000141	0.031	0.088	0.719
VTA1	BN1106_s2858B000111	0.31	0.117	0.363
ALIX	BN1106_s1871B000313	0.003	0.169	1.578
BROX	BN1106_s2963B000136	0.00057	0.101	0.722
**Small GTPases**				
Rab GDP dissociation inhibitor	BN1106_s605B000204	0.096	0.202	0.578
Rho GDP-dissociation inhibitor 2	BN1106_s4672B000098	0.0082	0.187	1.400
Rab-11B	BN1106_s844B000259	0.028	0.308	1.151
Rab-35	BN1106_s2985B000113	0.2	0.323	0.378
Rab GDP dissociation inhibitor alpha	BN1106_s605B000203	0.0063	0.143	0.000
Rab-3B	BN1106_s3297B000203	0.77	0.406	0.502
Ral-A	BN1106_s637B000246	0.021	0.000	0.868
Ras-related protein	BN1106_s2124B000372	0.23	0.000	0.427
Rho-Gap protein	BN1106_s789B000476	< 0.0001	0.067	0.000
Rab-18	BN1106_s2036B000204	0.0049	0.000	0.538
Rho GTPase Rac	BN1106_s4818B000076	0.12	0.000	0.238
**SNAREs**				
Synaptotagmin	BN1106_s3353B000056	0.084	0.121	0.397
Syntaxin 1A	BN1106_s1142B000130	0.003	0.078	0.713
Syntaxin-binding protein 1	BN1106_s1187B000336	0.93	0.081	0.077
Synaptotagmin	BN1106_s7334B000020	0.00014	0.000	0.353
Synaptotagmin-like protein 4	BN1106_s189B000725	0.92	0.044	0.040
SNAP-25	BN1106_s4176B000102	0.00077	0.000	0.728
SNAP-gamma	BN1106_s178B000373	0.95	0.167	0.161
**Vesicle transport**				
Syntenin-1	BN1106_s390B000196	0.0078	0.000	1.535
Syntenin-1	BN1106_s4740B000062	0.0089	0.000	1.703
Autophagy-related protein 9A	BN1106_s973B000182	0.0008	0.000	0.193
Pacsin	BN1106_s878B000298	0.0011	0.181	0.000
LAMP-1	BN1106_s2489B000362	0.01	0.040	0.296
VPS26	BN1106_s2566B000129	0.00027	0.094	0.000
**Membrane structure and remodelling**				
Annexin B2	BN1106_s500B000161	< 0.0001	1.295	0.573
Annexin	BN1106_s819B000365	0.00099	0.195	0.000
Annexin	BN1106_s3266B000046	0.02	0.149	0.973
Annexin	BN1106_s819B000364	0.033	1.568	1.022
Annexin	BN1106_s945B000218	0.032	0.623	0.992
Acid sphingomyelinase	BN1106_s1285B000159	0.34	0.690	0.976
Na^+^/H^+^ exchange regulatory cofactor	BN1106_s2296B000149	0.0013	1.892	0.000
SH3-domain GRB2-like endophilin	BN1106_s335B000427	0.23	0.399	0.750
EHD1 dynamin-like	BN1106_s2100B000128	0.86	0.057	0.000
Myoferlin	BN1106_s3585B000136	0.015	0.682	1.606
Otoferlin	BN1106_s3428B000062	0.00029	0.102	0.931
CD63 antigen	BN1106_s4560B000072	0.18	0.048	0.324
Tetraspanin-CD63 receptor	BN1106_s1657B000161	0.016	0.207	0.603

To validate the label-free quantitative proteomics analysis, equal amounts of the 15k and 120k EVs (10 μg) were probed with specific antibodies raised against selected surface proteins by immunoblotting. Consistent with our quantitative proteomics data, PDCD6IP, TSG101, RAL-A and CD63 receptor were all considerably enriched in 120k EVs (Fig 1C). The blots showed that aSMase and DM9-containing protein were found in both EV sub-types in agreement with the quantitative proteomics analysis; both fell below the significance threshold (*p* = 0.05) for enrichment in either EV sub-population (Fig 1B).

### Immunofluorescence confirms that the F. hepatica EV sub-populations have different cellular origins

**Fig 2:**
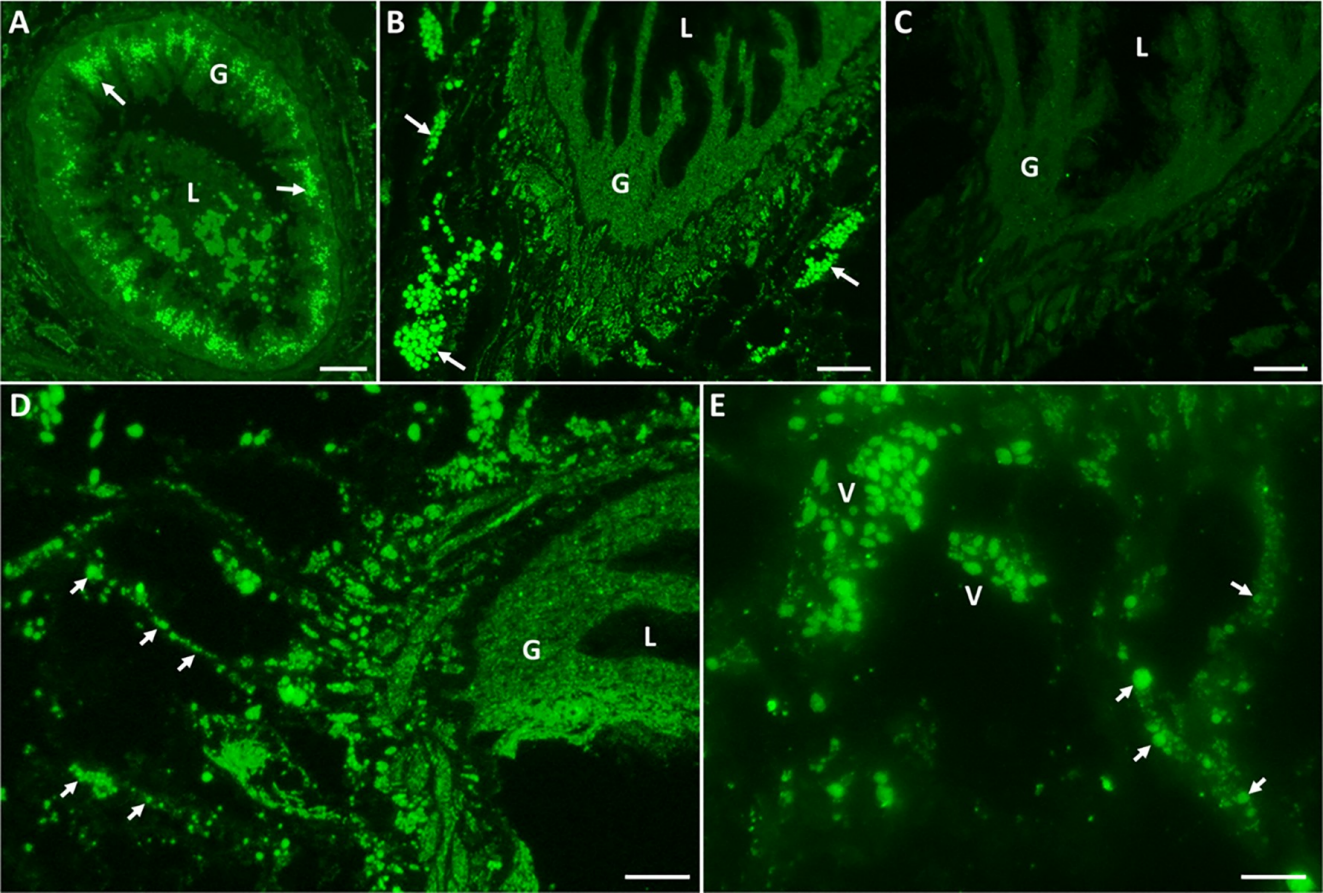
CD63 receptor-positive EVs are found in sub-gastrodermal regions of adult *F*. *hepatica*. Tissue sections of adult *F*. *hepatica* were probed with anti-cathepsin L (A) or anti-CD63 receptor antibodies (B, D-E) or with rabbit pre-immune serum (C). A. Specific cathepsin L immunoreactivity (arrows) can be seen within the gastrodermal cells (G) that line the parasite gut. L, gut lumen. B. Strong CD63 receptor immunolabeling is observed in vesicles that occur as distinct clusters (arrows) just beneath the gastrodermal cell layer. C. No specific immunofluorescence can be seen in these areas when sections were probed pre-immune control sera. D-E. CD63 receptor-positive vesicles (V) can be seen along duct-like structures (arrows) that converge upon the vesicle clusters in sub-gastrodermal regions of the adult fluke. Scale bars 25 μm (A-C) or 10 μm (D-E).

**Fig 3:**
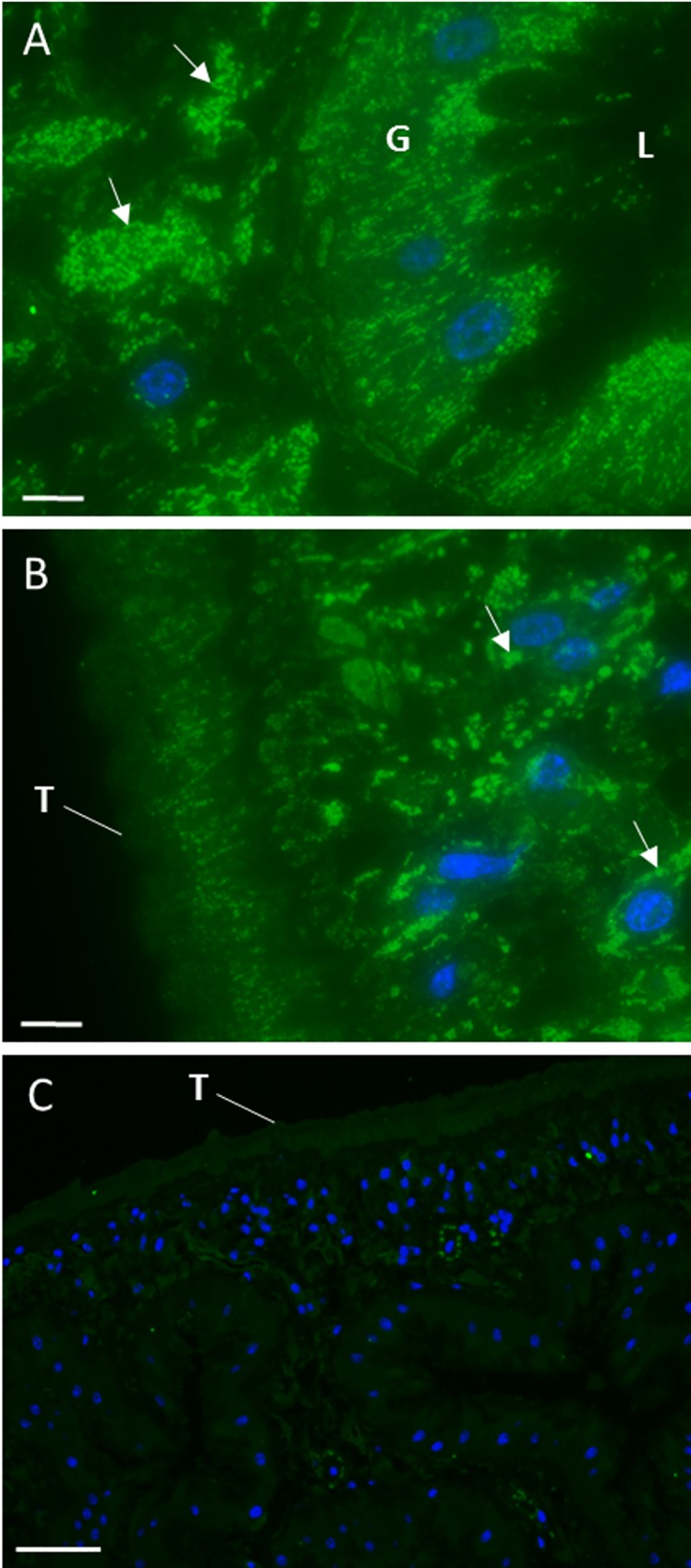
RAL-A-positive EVs are found in sub-gastrodermal regions and other structures of adult *F*. *hepatica*. Tissue sections of adult *F*. *hepatica* were probed with anti-RAL-A antibodies (A-B) or with rabbit pre-immune serum (C). A. Strong RAL-A immunolabeling is observed in vesicles that occur as distinct clusters (arrows) in extracellular locations just beneath the gastrodermal cell layer. Specific RAL-A immunoreactivity can be seen within the gastrodermal cells (G) that line the parasite gut. L, gut lumen. B. Faint immunofluorescence was observed throughout the tegumental syncytium (T) and in the underlying tegumental cell bodies (arrows). C. No specific immunofluorescence was observed when sections were probed with pre-immune control sera. Scale bars 50 μm (A) or 7.5 μm (B-C).

Adult *F*. *hepatica* tissue sections were probed with anti-sera raised against the *F*. *hepatica* CD63 receptor and RAL-A (as markers for the 120k EVs) or cathepsin L1 (as a marker for the 15k EVs) to determine the site of production/release of EVs from the parasite. We have previously shown that cathepsin L1 is specific for the 15k EVs [10] and, consistent with this, it was localised solely to vesicles distributed uniformly throughout the gastrodermal cells that line the fluke gut (Fig 2A). In contrast, clusters of CD63 receptor-positive vesicles were seen in distinct regions just beneath the gastrodermis (Fig 2B). At higher magnifications, the CD63 receptor-positive vesicles were observed within branched ducts leading from larger groups of vesicles and converging on the gastrodermal cell layer (Fig 2D and 2E). Like CD63, RAL-A also localised to clusters of vesicles lying just beneath the gastrodermis in extracellular locations (Fig 3A). Additional fluorescence (not seen with CD63) was observed in the gastrodermal cells (Fig 3A) whilst a faint signal was also detected in the tegumental syncytium and underlying tegumental cells (Fig 3B). No fluorescence was seen from either of these vesicle sub-populations when sections were probed with pre-immune sera (Figs 2C and 3C).

### Lectin microarray profiling of EVs and comparison to FhTeg profiles

**Fig 4:**
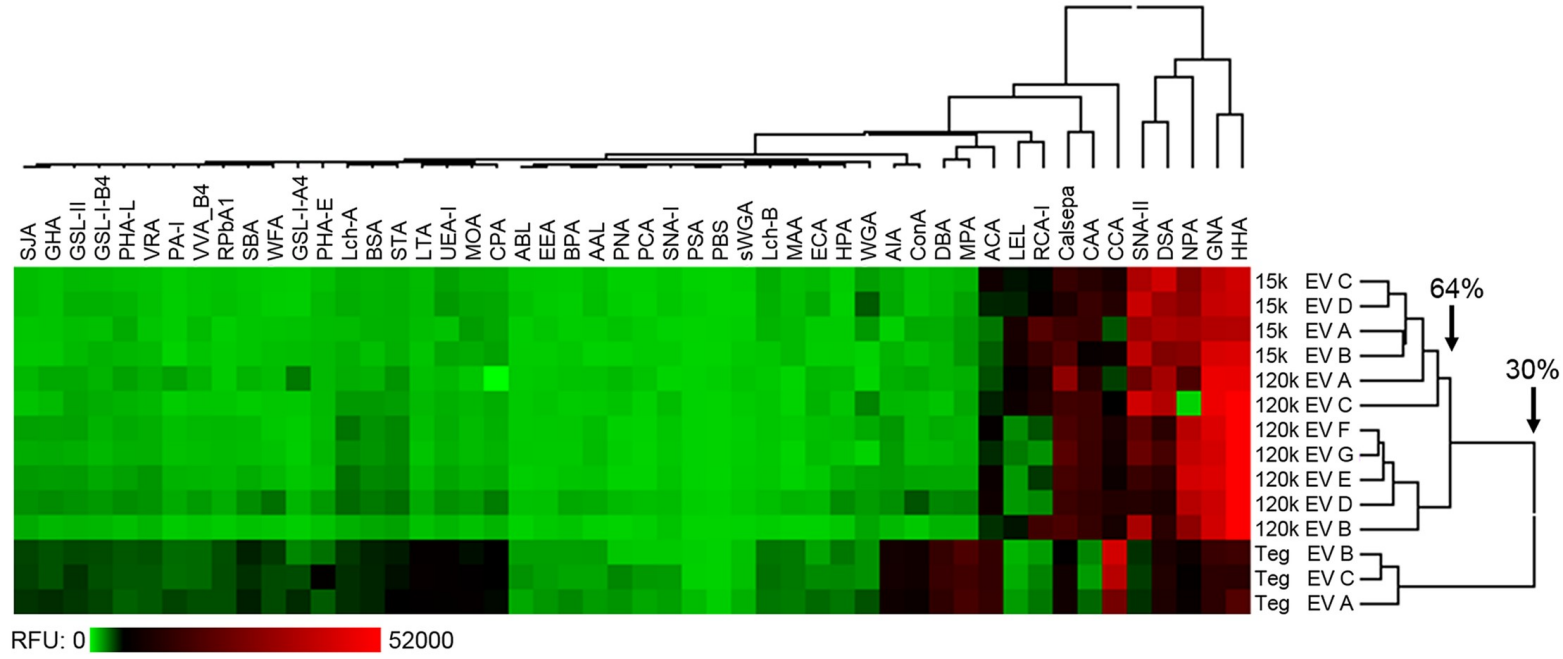
Comparison of 15k and 120k EV and tegument (Teg) mean lectin microarray responses. Heat map and two-dimensional hierarchical clustering of scale-normalized lectin microarray profile data for all technical replicates. Data depicted in heat map was scaled to fit a 0–30,000 RFU window and clustered by average linkage, Euclidean distance method.

A lectin microarray strategy was used to profile the major surface oligosaccharides of 15k and 120k EVs. Since vesicles were screened intact, this allowed surface structures to be assayed *in situ*. Accordingly, this makes lectin microarray a particularly powerful method for identifying molecules as they would be displayed to the host microenvironment *in vivo*. Profiles obtained for intact *F*. *hepatica* EVs include contributions from not only the surface and transmembrane glycoproteins, but also glycolipids and glycosaminoglycan components. The 15k and 120k EVs generated an almost identical lectin-binding profile that was dominated by strong signals (>5000 RFU above local background) from eleven lectins: SNA-II, ACA, DSA, LEL, Calsepa, NPA, GNA, HHA, CCA, RCA-I and CAA (Fig 4, S3 Fig). The remaining lectins demonstrated only low-level interactions with the EVs. In contrast, lectin microarray profiles obtained for extracted tegument proteins (FhTeg) bound to a broad range of lectins (Fig 4, S3 Fig) albeit at low intensity for the majority. Two-dimensional, unsupervised hierarchical clustering of the scale-normalized replicate profile data resulted in a clear division of the EVs vs FhTeg samples (Fig 4), which indicated that they each displayed either different combinations and/or abundances of glycan structures. In general agreement with the glycoanalysis reported for *F*. *hepatica* tegumental surface preparations [19], the lectin profiles for both EV sub-populations suggested the presence of high mannose *N*-linked structures on the EV surface, through binding of Calsepa, NPA, GNA and HHA, as well as more diverse and complex structures (potentially components of *N*- or *O*-linked protein glycosylation or glycolipid structures) as suggested by binding of SNA-II, ACA, DSA, LEL, RCA-I and CAA.

### Establishment of carbohydrate-mediated EV-lectin binding

**Fig 5:**
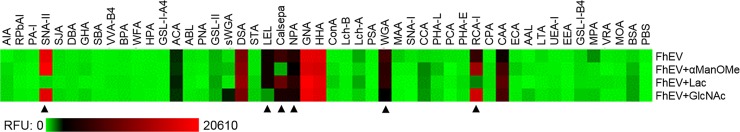
Competitive inhibition of lectin binding on the 120k EV surface.Arrowheads indicate significant (*p* ≤ 0.05) mean lectin microarray response changes imparted by competitive inhibition with 50 mM final concentrations of Lac, αManOMe or GlcNAc as indicated.

To establish that the binding between the lectins and the EVs was indeed carbohydrate mediated, selected sugars (Lac, αManOMe, GlcNAc) were tested as competitive inhibitors of EV binding. Since the lectin-binding profiles of the 15k and 120k EVs were extremely similar, only the 120k EVs were used in subsequent microarray experiments. In the presence of Lac, significant inhibition was observed at lectins SNA-II (-98%, *p* < 0.01) and RCA-I (-89%, *p* < 0.05) (Fig 5, S4 Fig). Significant lectin-binding inhibition was observed in the presence of αManOMe at Man-binding lectins Calsepa (-92%, *p* < 0.01) and NPA (-65%, *p* < 0.05) (Fig 5, S4 Fig). Man-binding lectins HHA and GNA were not inhibited by αManOMe, but this is consistent with previous reports showing the dependence of HHA and some other lectins upon multiple residues in an oligosaccharide for specific binding [23]. With GlcNAc, significant inhibition was observed at lectins LEL (-80%) and WGA (-77%) (both *p* < 0.01) (Fig 5, S4 Fig). Curiously, signals increased at CAA for the EVs in the presence of Lac, αManOMe and GlcNAc (Fig 5, S4 Fig). Similarly, signals increased for RCA-I in the presence of αManOMe and GlcNAc, leading to the speculation of some stabilizing effect generated by the inclusion of the soluble sugars–either enabling more accessible presentation of the EV surface glycans or the binding of EV surface-presented adhesion molecules to the protein or carbohydrate components of the lectins to which binding intensity was increased.

### Modulation of EV-lectin interactions by glycosylation-specific hydrolases

**Fig 6:**
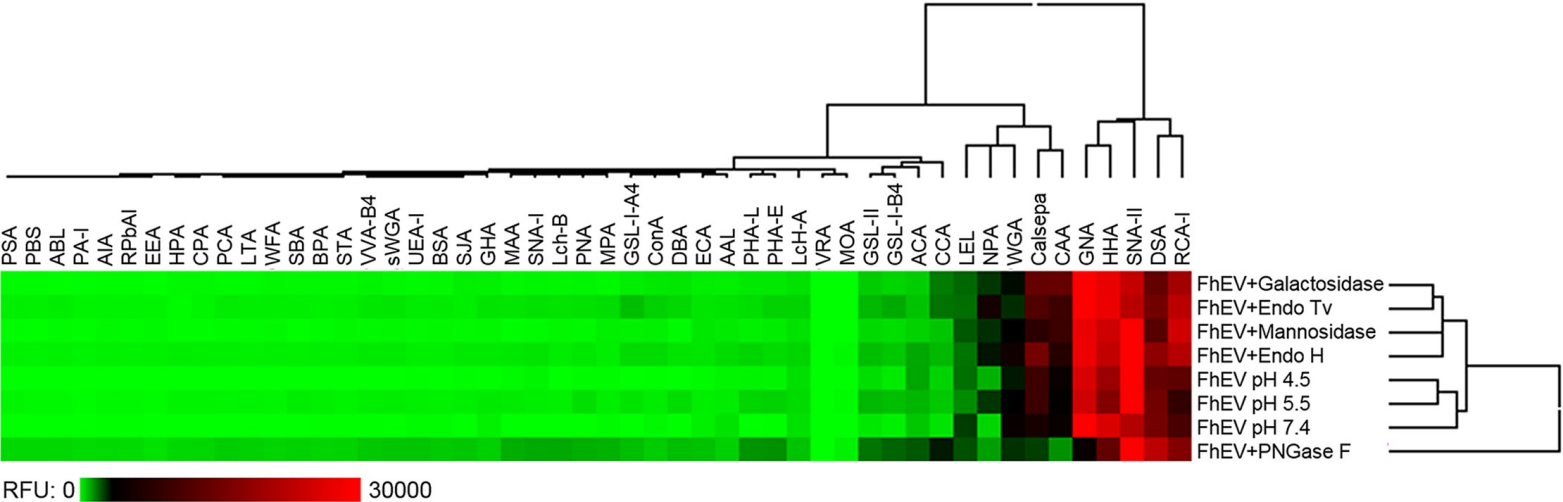
Comparison of the effects of glycosidase treatment on 120k EV lectin-binding profiles. Heat map of 120k EV mean lectin microarray profiles imparted by treatment with *exo*-glycosidases, *endo*-glycosidases or glyco-amidase in comparison with their respective untreated, pH-adjusted controls. Mean data derived from total intensity mean adjusted replicate data (mannosidase, galactosidase (n = 5); PNGase F, Endo Tv, Endo H (n = 4); non-enzyme controls adjusted to pH 4.5, pH 5.5, pH 7.4 (n = 3)). All data subjected to two-dimensional hierarchical clustering by average linkage, Euclidean distance method.

*Exo*- and *endo*-glycosidase treatments were set up with PKH26-labelled EVs prior to their profiling by lectin microarray (Fig 6; S5 Fig; S6 Fig). Lectin microarray profiles for EVs treated with β-galactosidase demonstrated a reduction in binding intensity at SNA-II (-25%, *p* < 0.01) and reductions at a number of lectins which demonstrated low intensity EV binding (Fig 6, S5 Fig). ACA, a lectin which recognizes β-linked Gal terminations with a marked preference for Gal-β(1→3)-GalNAc, demonstrated a strong reduction in signal (-57%) after EVs were treated with β-galactosidase (Fig 6, S5 Fig). However, RCA-I demonstrated a modest increase in binding (+14%) instead of the expected decrease (S5 Fig). A number of minor response changes were also observed across the profile (S5 Fig).

Following from the previous reports of high mannose structures on *F*. *hepatica* tegumental surface proteins [19,24], it was anticipated that α-mannosidase would have a substantial impact on EV binding at a subset of lectins that bind to such structures. Surprisingly, however, only Calsepa and GNA demonstrated modest and insignificant reductions in signal following α-mannosidase treatment of the FhEVs (Fig 6, S5 Fig). NPA showed an increase in binding after α-mannosidase treatment (but with high variability across replicates) whilst HHA demonstrated a marginal increase in signal (Fig 6, S5 Fig). Given that incubation conditions were stabilized to favour the enzyme, this result was highly unexpected.

The *endo*-β-*N*-acetylglucosaminidases Endo H and Endo Tv have similar specificity for high mannose-type *N*-linked oligosaccharides [21]. Here, these comparable enzymes produced similar changes in overall EV lectin-binding profiles, although these were very modest (Fig 6). Endo H produced no significant profile changes for the EVs, except for a substantial increase in binding at the Type-II *N*-acetyllactosamine (LacNAc)-specific RCA-I (+51%, *p* < 0.05) which was mirrored by Endo Tv at RCA-I (+56%) (Fig 6, S6 Fig). Other lectins which bind to complex and hybrid, *N*-linked oligosaccharides, such as PHA-E, PHA-L, PCA and CPA, did not demonstrate increases in binding after the theoretical removal of the number of high mannose structures on the EV surface (although CAA did demonstrate the trend of increased binding observed with most of the treated EVs) (Fig 6, S6 Fig).

In sharp contrast, to the *exo*- and *endo*-glycosidases, the glyco-amidase PNGase F drastically remodelled the EV lectin microarray profile (Fig 6 and S6 Fig). As the incubation conditions were chosen to keep the vesicles intact (no detergents or reducing agents), access to the amide bond formed between peptides and attached *N*-linked oligosaccharides may still have been somewhat restricted. Nonetheless, the changes in profile suggested removal of a high proportion of the high-mannose glycans and potentially the un-masking of other, presumably non-*N*-linked structures which were then able to interact with a number of lectins (Fig 6, S6 Fig). Specifically, high-mannose binding lectins Calsepa (-45%, *p* < 0.01), GNA (-67%, *p* < 0.01) and HHA (-35%, *p* < 0.05) demonstrated substantial reductions in intensity with PNGase F-treated EVs in comparison to the pH 7.4 (no enzyme) negative control (Fig 6, S6 Fig). Lectins ACA, PNA, GS-I-A4, DSA, LcH B, MAA, SNA-I, CCA, PHA-L and GS-I-B4 all demonstrated some degree of signal increase with PNGase F-treated EVs in comparison to their untreated counterparts (Fig 6, S6 Fig) suggesting an unmasking of structures through the removal of longer *N*-linked oligosaccharides.

### Internalisation of F. hepatica EVs by macrophages is blocked by glycosidase treatment

**Fig 7:**
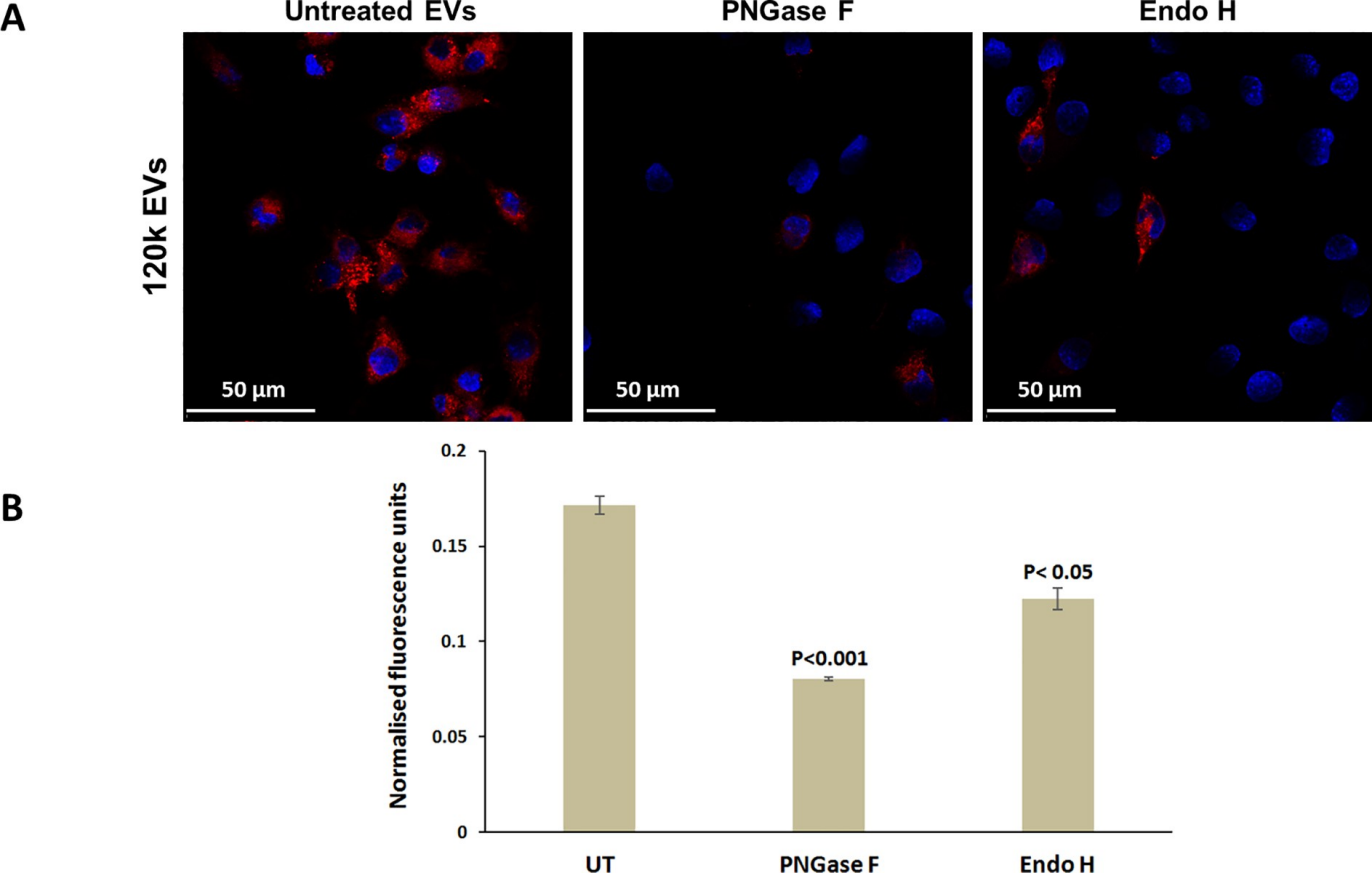
De-glycosylation of *F*. *hepatica* EVs blocks their internalisation by host macrophages. (A) RAW264.7 macrophages were incubated with PKH26-labelled 120k EVs for 3 h at 37°C and the cells were analysed by confocal microscopy. (B) Pre-treatment of labelled EVs with the glycosidases PNGase F and Endo H significantly reduced the uptake of the EVs when measured as fluorescence intensity relative to cells incubated with untreated EVs.

To investigate the role of carbohydrates displayed on the EV outer surface in vesicle uptake, PKH26-labelled *F*. *hepatica* EVs were pre-incubated with PNGase F or Endo H and added to RAW246.7 macrophages in culture. Non-modified EVs were readily taken up by the cells and could be seen as discrete areas of red fluorescence evenly dispersed throughout the cytoplasm (Fig 7A; S7 Fig). Treatment of cells with cytochalasin D (a blocker of actin polymerisation and endocytosis pathways) inhibited EV internalisation confirming that uptake is an active process (S8 Fig). Similarly, cells incubated with the glycosidase-treated EVs showed reduced red fluorescence suggesting that de-glycosylation of the EVs blocked their uptake by macrophages (*p* range < 0.001 to < 0.05; Fig 7B). Treatment of EVs with *exo*-glycosidases (α1–2,3 mannosidase and β1–4 galactosidase) did not have any effect on EV uptake by macrophages (S9 Fig) in agreement with the inability of these enzymes to significantly alter the profile of carbohydrates displayed on the EV surface.

### Internalisation of F. hepatica EVs by macrophages is enhanced in the presence of host antiserum and antibodies prepared against specific EV surface proteins

**Fig 8:**
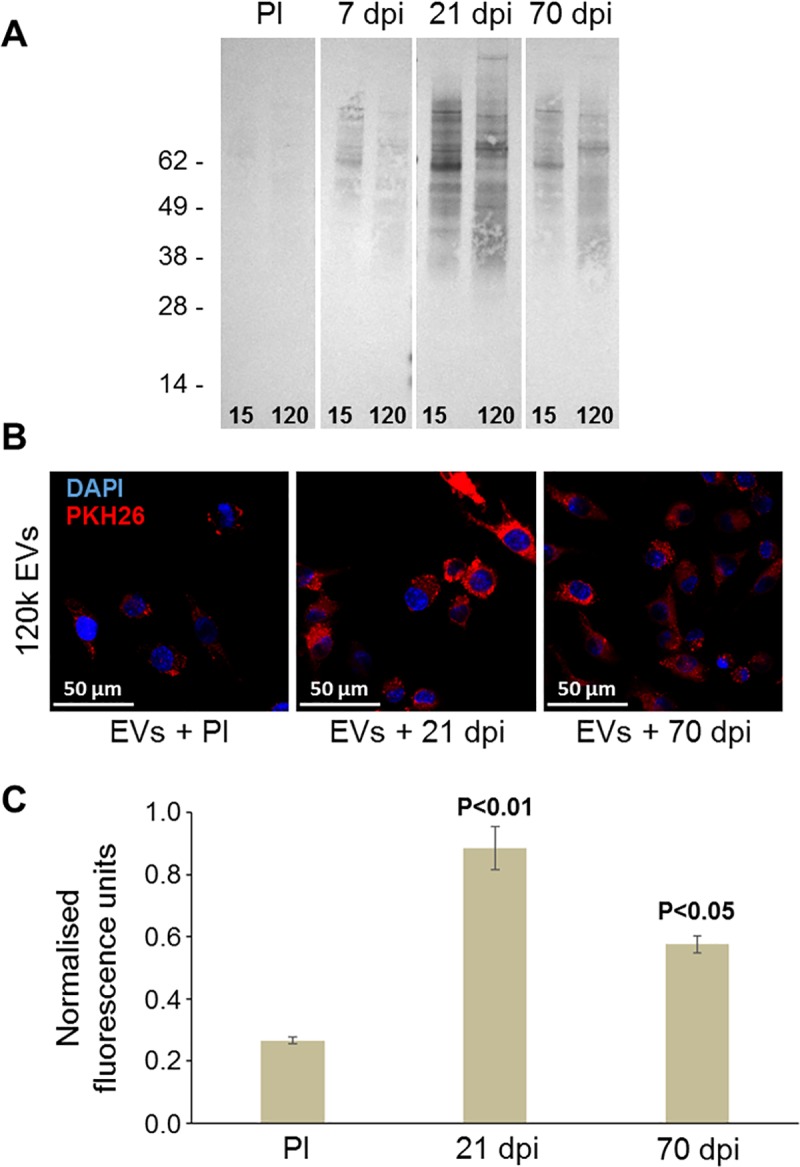
Host antiserum enhances internalisation of *F*. *hepatica* EVs by macrophages. (A) Immunogenicity of *F*. *hepatica* EVs in *F*. *hepatica*-infected rats. Equal amounts (10μg total protein) of 15k and 120k EVs were analysed by Western blot using pre-infection (PI) sera and serum samples taken 7, 21 and 70 days post-infection. (B) RAW264.7 macrophages were incubated with PKH26-labelled 120k EVs for 3 h at 37°C and the cells were analysed by confocal microscopy. EVs pre-incubated with pre-infection rat serum were internalised by the macrophages as shown by the punctate red fluorescence observed throughout the cytoplasm. However, when EVs were pre-treated with 21-day rat serum the internalisation the EVs increased considerably. A similar, but less dramatic, effect was observed when EVs were pre-treated with the 70-day serum. Nuclei were stained with DAPI (blue). (C) These effects were statistically significant when measured as fluorescence intensity relative to cells treated with EVs pre-incubated with pre-infection serum.

We next examined the role of surface-exposed proteins in vesicle uptake by host cells. Western blot analysis, using serum samples taken from rats, experimentally infected with *F*. *hepatica*, showed that both the 15k and 120k EVs induced a host antibody response as early as 7 days post-infection. This response intensified as the infection progressed with the strongest response seen at 21 days post-infection which corresponds to the migration of the fluke through the liver parenchyma in the rat model (Fig 8A). We then tested the effect of these host antibodies on the uptake of *F*. *hepatica* EVs by macrophages. The 15k and 120k EVs were stained with the lipophilic dye PKH26 and incubated with rat pre-infection serum or sera taken at 21 days and 70 days post-infection. Unbound antibodies were removed by centrifugation and the EVs were incubated with RAW246.7 macrophages for 3 h at 37°C before the cells were analysed by confocal microscopy. When pre-treated with rat pre-infection serum, the EVs were internalised by the macrophages as shown by discrete, punctate regions of red fluorescence that were observed throughout the cytoplasm (Fig 8B; S7 Fig). However, when pre-treated with 21-day post-infection rat serum the internalisation of the 120k EVs increased significantly (*p* < 0.01; Fig 8C). A similar, but less dramatic, effect was observed when EVs were pre-treated with the 70-day serum (*p* < 0.05; Fig 8C) consistent with the level of antibody response seen by Western blotting (Fig 8A). Similar effects on uptake were observed using the 15k EVs (S7 Fig).

**Fig 9:**
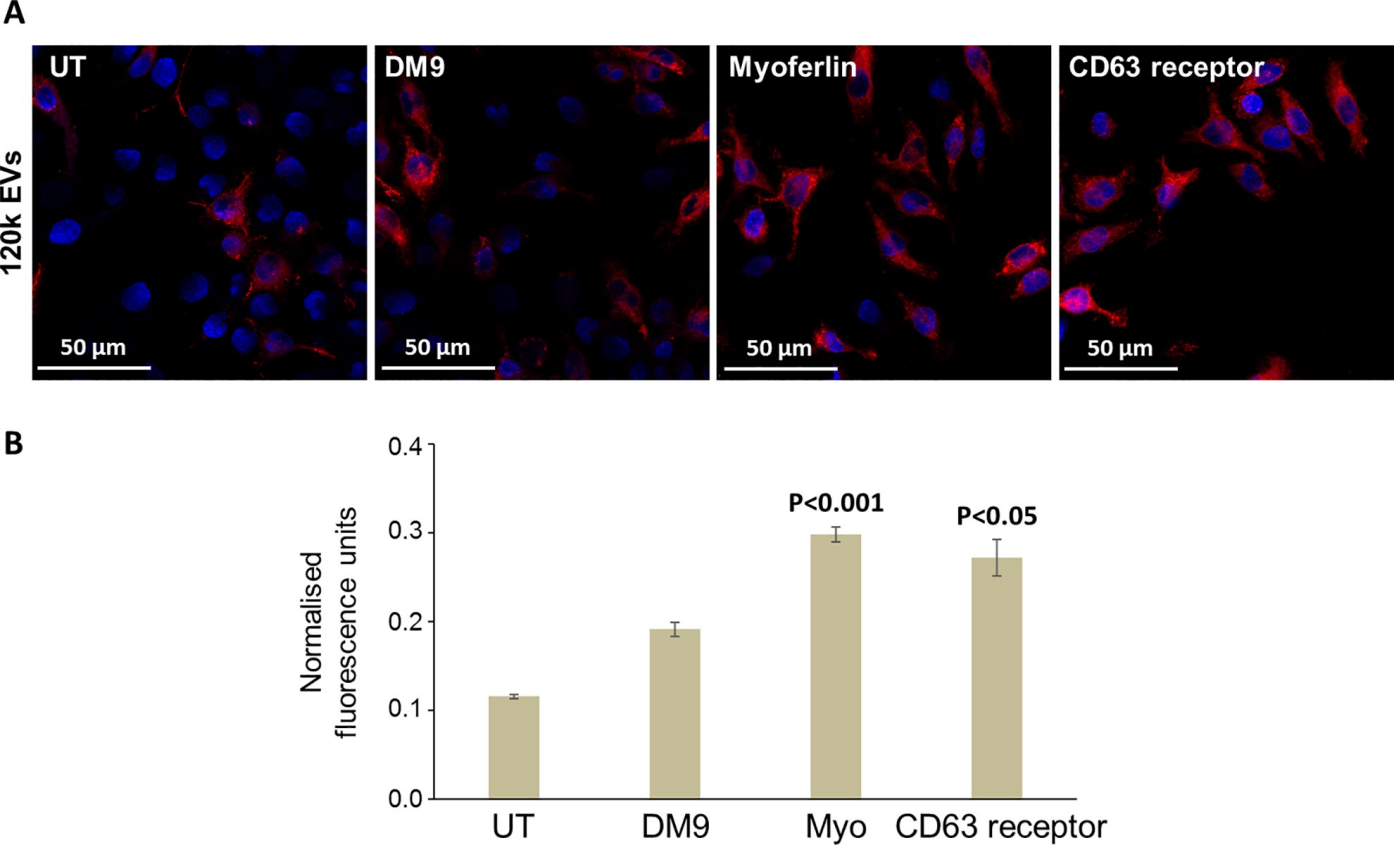
Antibodies raised against specific *F*. *hepatica* EV surface proteins also influence uptake by macrophages. (A) RAW264.7 macrophages were incubated with PKH26-labelled 120k EVs for 3 h at 37°C and the cells were analysed by confocal microscopy. Pre-treatment of labelled EVs with antibodies raised against specific *F*. *hepatica* proteins (DM9, myoferlin and CD63 receptor) resulted in a greater level of internalisation by macrophages compared to control EVs (left panels). (B) Internalisation of myoferlin- and CD63 receptor-treated EVs was statistically significant when measured as fluorescence intensity relative to cells incubated with untreated EVs.

To investigate the functional role of individual EV surface proteins, the PKH26-labelled vesicles were incubated with antibodies raised against specific surface proteins. These were selected from the 380 proteins identified following biotin pulldown as either potential ligands for receptor-mediated endocytosis (CD63 receptor and DM9-containing protein) having roles in EV biogenesis and trafficking (aSMase) or suspected fusogenic properties (myoferlin). The antibody-treated EVs were again added to RAW264.7 macrophages for 3 h at 37°C and the cells were analysed by confocal microscopy (Fig 9A). As observed with the rat serum, pre-treatment of labelled 120k EVs with antibodies raised against DM9-containing protein, myoferlin (*p* < 0.001), and CD63 receptor (*p* < 0.05) resulted in a greater level of internalisation by macrophages compared to control EVs (Fig 9B). Similar effects on internalisation were observed when 15k EVs were pre-incubated with antibodies raised against DM9-containing protein, myoferlin and aSMase (S7 Fig).

## Discussion

There is considerable interest in EVs as vehicles for the transfer of parasite-derived molecules to host cells. Many researchers have successfully used proteomics techniques to identify their resident proteins [9,10,25–27] and, therefore, we have a good working knowledge of the EV cargo proteins and are beginning to understand those involved in EV biogenesis and trafficking pathways [10,13]. By contrast, little is known about the molecules that modify the surface of parasite EVs. Since these molecules likely make contact with receptors on the host cell plasma membrane (initiating their endocytosis), they would be obvious targets for interventions that aim to prevent parasite EV-host cell interaction. In this study we have profiled the proteins and oligosaccharides exposed on the surface of the 15k and 120k *F*. *hepatica* EVs which has revealed a great deal about how EVs are formed and trafficked by this parasite but also how they interact with recipient host cells.

Cells release different populations of EVs, which may either bud directly from their plasma membrane (microvesicles) or derive from the endosomal pathway (exosomes). In keeping with our previous studies, analysis of the 120k *F*. *hepatica* EVs revealed an enrichment in molecules of the endosomal sorting complex required for transport (ESCRT) pathway, the main driver of exosome formation in mammalian cells [28], suggesting an endosomal origin for these vesicles [10]. These included members of the ESCRT-III complex and the Vps4 ATPase responsible for intra-luminal vesicle budding and abscission, respectively, as well as the ESCRT-associated proteins ALIX and syntenin. Other proteins that provide further support for an endosomal origin of the parasite 120k EVs include STAM-binding protein and TSG101, (ESCRT-0 and ESCRT-I components, respectively), as well as a number of SNAREs and Rab GTPases. These molecules are involved in vesicle trafficking by bringing opposing membranes together and catalysing their fusion [29] and have important roles in exosome biogenesis and secretion [30]. Their presence on the surface of *F*. *hepatica* EVs may simply be a remnant carried over from their endosomal origins but they could also mediate tethering and fusion of the parasite EVs with membrane-bound organelles following their endocytosis by host cells.

The tetraspanin annotated as CD63 receptor is a trematode-specific membrane protein [10] whilst RAL-A is a small GTPase required for MVB biogenesis and EV release in *Caenorhabditis elegans* [31]. Since both are highly enriched in *F*. *hepatica* 120k EVs, compared to the 15k EV population, we employed anti-CD63 and anti-RAL-A antibodies in immunofluorescence studies to locate the origin of the 120k EVs in adult parasites. Surprisingly, distinct clusters of vesicles, showing both CD63 receptor and RAL-A immunostaining were observed just beneath the gastrodermis. CD63-labelled vesicles were also seen within “Y-branched” ducts, characteristic of the liver fluke protonephridial (excretory) system described many years ago by Pantelouris and Threadgold [32] whilst RAL-A vesicles lay in similar extracellular locations. Interestingly, the ducts appeared to converge upon the gastrodermis suggesting that they may drain into the gut lumen, a scenario also deemed possible for the protonephridial system of free-living planarians [33]. RAL-A is a multifunctional GTPase with varied roles in vesicle trafficking [34] so it was perhaps not surprising that it was found associated with the tegument and gut, both of which are major sites of secretion in the parasite.

The 15k EVs were localised to the gastrodermal cells that line the parasite gut in keeping with our previous ultrastructural observations [10]. This tissue-specific distribution, together with the enrichment of saposin (previously shown to lyse host erythrocytes; [35]) and proteases, such as cathepsin L1, cathepsin L2 and an aspartic protease, that can efficiently degrade host haemoglobin in *F*. *hepatica* and other trematodes [36,37] strongly suggests a digestive function for the 15k EVs. Following ultrastructural examination of the *F*. *hepatica* gut, Halton [38] suggested that the numerous secretory vesicles held with the gastrodermal cells are released into the lumen of the gut upon rupture of the apical plasma membrane. The stimulus for this periodic mass release appears to be the presence of food in the gut and the enrichment of numerous calcium-binding/sensing molecules (including *F*. *hepatica* calcium-binding protein 4, calmodulins, calreticulin and EF-hand domain proteins) in the 15k EVs suggests that calcium signalling pathways may regulate this process.

We previously used trypsin-shaving (and subsequent sequential membrane extraction) to identify surface-exposed and membrane-associated proteins of intact *F*. *hepatica* 120k EVs [10]. Using the current biotinylation approach, we identified a further 111 surface proteins that were not found in the earlier study. Sixty of these (54%; dominated by vesicle trafficking proteins and pumps/transporters) are reported to be membrane proteins based on GO terms or experimental evidence in the literature. A number of cytosolic proteins and digestive enzymes, such as cathepsin B and L peptidases, were also found amongst the EV surface proteins. Although unexpected, it is not unusual for cytoplasmic/soluble proteins to be found on the EV surface. Indeed, a recent study showed that 51.1% of proteins found on the surface of EVs derived from mast cells were cytosolic [39]. Whilst these could be artefacts we applied very stringent protein acceptance criteria in order to eliminate non-specific contaminants during affinity-purification of biotinylated EV proteins. A more likely explanation, in agreement with Cvjetkovic et al. [39], is that the cytosolic/soluble proteins adhere to the surface and co-purify with the EVs. Since cathepsins and EVs are co-secreted from the fluke *in vivo* this association could be real. In our earlier study of *F*. *hepatica* EVs we observed that most cathepsins were recovered from carbonate washes of the EV membrane [10] suggesting that they make electrostatic interactions with the EV surface and may only adhere upon secretion from the parasite.

Whilst our proteomics analysis showed clear enrichment of proteins associated with the 15k and 120k EV sub-populations, it is likely that some level of heterogeneity will occur due to the ultracentrifugation method used to isolate them. However, Kowal et al. [40] found that molecules considered to be “classic” exosome markers, such as CD63, are also present on larger vesicles secreted by human dendritic cells (i.e. microvesicles that are typically recovered following 10–15,000 x g centrifugation of cell culture media). Thus, it could be a worthwhile exercise to exploit antibodies to a wider array of molecules to classify the various cellular/tissue origins of helminth EVs and to track their trafficking and release.

In addition to characterising the *F*. *hepatica* EV surface proteome, we used recently developed methods [20] to profile the major oligosaccharides displayed on the EV surface as a first step towards understanding the role of glycoconjugates in the host-parasite relationship. The approach of using intact EVs allowed a more biologically relevant scenario for profiling ligand/receptor arrangement on the surface of membranes compared to profiling distinct molecules in isolation. Comparative lectin-binding data demonstrated that the *F*. *hepatica* EV and tegumental (FhTeg) profiles are distinct from one another; the EV profiles are more ‘punctuated’ by strong responses at fewer lectins whilst the extracted FhTeg protein population bound to a greater number of different lectins. These findings suggest that either *F*. *hepatica* EVs display less overall oligosaccharide diversity on their surface or that select, extended structures dominate the EV surface and mask shorter underlying structures. Overall, these data, together with our immunofluorescence studies, support our idea that both the 15k and 120k EVs are selectively synthesised and secreted by the parasites and are not simply derived from shedding or blebbing of the surface tegument [10,13].

In this work, modulation of EV-lectin binding patterns was possible through both competitive inhibition with sugars and by specific enzymatic manipulation. Several of the interactions between the lectins and EVs could be selectively and sharply inhibited with αManOMe, GlcNAc or Lac, demonstrating that they were, indeed, mediated by carbohydrates present on the *F*. *hepatica* EV surface. Some evidence of carbohydrate-specific enzymatic sensitivity was observed for the intact EVs, but the changes generated, even after extended exposure to select enzymes, were relatively modest except for those following treatment with the glyco-amidase PNGase F.

Lectin microarrays also demonstrated that the *F*. *hepatica* EV surface is predominated by mannose-bearing glycoconjugates. Despite this, exposure to *exo*-α-mannosidase, a broad-spectrum enzyme that hydrolyses Man residues connected in α-(1→2, 3 and 6) linkages, only subtly altered the binding profile of the EVs. The lack of substantial alteration of the lectin microarray profile by Endo Tv and Endo H was also unexpected since PNGase F produced a significant revision of the EV-lectin microarray profile, even at lectins which are reported to recognize mannose and mannose-containing oligosaccharides. Collectively, these studies suggest a condition of the presentation of high-mannose, *N*-linked oligosaccharides that does not allow hydrolysis by the selected *endo*-glycosidases but does permit their removal with the glyco-amidase. A growing number of studies suggest that unusual or truly novel oligosaccharide structures (including di- and tri-fucosyl substitution of GlcNAc residues in the chitobiose core of *N*-linked glycans or inclusion of phophorylcholine or fucosylation and/or xylosylation at the non-reducing end) are common features of helminth glycomes [41–43] which may explain the resistance of *F*. *hepatica* EV surface proteins to *exo*- and *endo*-glycosidases that are commonly used to modify mammalian structures. The biological significance of this remains to be elucidated, although having *N*-linked oligosaccharides that are resistant to degradation may be beneficial to the parasite by allowing prolonged biological activity of the EVs in the parasite-host microenvironment.

The primary mechanism for uptake of parasite EVs by recipient cells appears to be via endocytosis [25,44]. However, an alternative fate for parasite-derived EVs was recently described for the protozoan *Trypanosoma brucei* whereby their cargo was delivered directly into the host cell cytoplasm following fusion of the EVs with the plasma membrane [45]. Although, this mechanism of delivery has yet to be reported for helminth-derived vesicles it was of interest that we discovered several molecules on the surface of *F*. *hepatica* EVs with potential fusogenic properties, i.e. the ability to merge phospholipid bilayers. Two of these, myoferlin (enriched in the 120k EVs) and EHD1 (dynamin-like; unique to 15k EVs), have been shown to promote fusion of myoblast plasma membranes [46,47], whilst myoferlin-depleted mammalian exosomes are taken up less efficiently by target cells [48]. A third molecule, GAPDH (unique to the 120k EVs; [10]), in addition to its established role in glycolysis, also has potent fusogenic properties and can merge plasmenylethanolamine-containing phospholipid bilayers [49,50]. Recent work has shown that the EVs released by the nematode *Heligmosomoides polygyrus* are enriched in similar specialised phospholipids [51].

Conceivably, EV surface-expressed fusogenic molecules, or other molecules that serve as ligands for host cell surface receptors, could be targeted to disrupt parasite EV-host cell interactions. Indeed, treatment of EVs derived from adult *Opisthorchis viverrini*, a trematode parasite related to *F*. *hepatica*, with anti-tetraspanin antibodies blocks their uptake by cholangiocytes *in vitro* [25] which provides proof-of-concept for this strategy. However, we observed that pre-treatment of both the *F*. *hepatica* 15k and 120k EVs with sera obtained from infected rat hosts significantly enhanced their uptake by macrophages *in vitro* with the level of internalisation correlating with the immunoreactivity of the serum against the EVs (21 days post-infection > 70 days post-infection). Enhanced uptake was also observed using purified antibodies raised against several specific EV surface proteins including DM9-containing protein, myoferlin, aSMase and CD63 receptor (not a direct homolog of the CD63 targeted in *O*. *viverrini*). These unexpected findings are in agreement with a recent study in which host antisera increased the uptake of *H*. *polygyrus* EVs by macrophages [44], presumably via opsonisation. The authors suggested that FcR-mediated uptake directs the parasite EVs into lysosomes where they are presumably degraded thus losing any functional effects on host cells. Whilst this may indeed be an appropriate host defence strategy, aimed at disarming the parasite’s immunomodulatory capabilities, delivery of some *F*. *hepatica* molecules to the endolysosomal system may, in fact, benefit the parasite; e.g. we have shown that HDM impairs antigen processing by macrophages via inhibition of lysosomal vATPase [6] and cathepsin L1 (which is resistant to lysosomal degradation) degrades TLR3 within the endosome [5]. Thus, caution should be taken when designing potential EV blocking agents as these may work with some, but not all, host cell types.

Here we have employed a novel approach to provide a comprehensive analysis of the proteins and oligosaccharides exposed on the surface of two sub-populations of *F*. *hepatica* EVs and demonstrated their importance in interactions with host cells. As our knowledge of the biological effects and practical uses of parasite EVs continues to grow, our findings may be used to direct the selection of anti-parasite vaccine candidates or to aid design of agents that may be used therapeutically to block parasite-derived EV function.

## Supporting information

S1 Table Proteins identified on the surface of *F. hepatica* EVs by mass spectrometry. (XLSX)

S1 Fig Surface biotinylation of *F. hepatica* EVs and streptavidin pulldown workflow. *F*. *hepatica* 15k and 120k EVs were incubated with a non-permeable biotin reagent. The EVs were solubilised with detergent and the biotinylated surface proteins recovered using a streptavidin affinity column.
Western blot with streptavidin conjugated with alkaline phosphatase showing the profile of biotinylated protein after sequential extraction. E1-E3, represent each extraction step. Biotinylated proteins are purified with high efficiency after the pull-down with streptavidin agarose beads (B) whilst non-biotinylated proteins are lost in the flow-through (S).
(TIF)

S2 Fig Enrichment of GO terms (A) and protein domains (B) for the proteins identified on the surface of the 15k and 120k EVs.
(TIF)

S3 Fig Bar chart comparing relative lectin microarray responses generated for 15k and 120k EVs and tegument samples. Data subjected to total intensity mean normalization. Error bars represent +/- one standard deviation.
(TIF)

S4 Fig Bar charts detailing competitive inhibition of lectin binding on the 120k EV surface. 120k EV mean lectin microarray response changes imparted by competitive inhibition with 50 mM final concentrations of A. Lac, B. αManOMe and C. GlcNAc. Error bars represent +/- one standard deviation based on three technical replicates. Significance of inhibition * *p* ≤ 0.05; ** *p* ≤ 0.01.
(TIF)

S5 Fig Effect of *exo*-glycosidase treatment on lectin-binding at the EV surface. A. 120k EV mean lectin microarray responses imparted by *exo*-glycosidase treatment with (A) β-galactosidase (n = 5) and (B) α-mannosidase (n = 5) and their respective pH-matched controls (controls n = 3). Error bars represent one +/- standard deviation.
(TIF)

S6 Fig Effect of *endo*-glycosidase treatment on lectin-binding at the EV surface. 120k EV mean lectin microarray responses imparted by treatment with *endo*-glycosidases A. Endo H (n = 4) and B. Endo Tv (n = 4) and glyco-amidase C. PNGase F (n = 4) and their respective pH-matched controls (all n = 3). Error bars represent +/- one standard deviation.
(TIF)

S7 Fig Uptake of 15k EVs by macrophages. RAW264.7 macrophages were incubated with PKH26-labelled 15k EVs for 3 h at 37°C and the cells were analysed by confocal microscopy. (A) Pre-treatment of labelled EVs with the glycosidases PNGase F and Endo H considerably reduced their uptake. (B) Pre-treatment of labelled EVs with 21-day rat serum increased the internalisation of the EVs considerably. A similar, but less dramatic, effect was observed when EVs were pre-treated with the 70-day serum. (C) Pre-treatment of labelled EVs with antibodies raised against specific *F*. *hepatica* proteins (DM9, myoferlin and aSMase) resulted in a greater level of internalisation by macrophages compared to control EVs (left panel).
(TIF)

S8 Fig Uptake of 120k EVs by macrophages is inhibited by cytochalasin D. RAW264.7 macrophages were incubated with PKH26-labelled 120k EVs for 3 h at 37°C and the cells were analysed by confocal microscopy. Co-incubation of cells with cytochalasin D (2μg/ml) inhibited uptake of EVs.
(TIF)

S9 Fig Uptake of 120k EVs by macrophages is not effected by *exo*-glycosidases. RAW264.7 macrophages were incubated with PKH26-labelled 120k EVs for 3 h at 37°C and the cells were analysed by confocal microscopy. Pre-treatment of labelled EVs with the *exo-*glycosidases α1–2,3 mannosidase and β1–4 galactosidase had no effect on the uptake of the EVs.
(TIF)
