# Supplementary figures and images for: Surface molecules of extracellular vesicles secreted by the helminth pathogen Fasciola hepatica direct their internalisation by host cells
Source: PLoS Negl Trop Dis. 2019 Jan 18;13(1):e0007087. doi: 10.1371/journal.pntd.0007087 (PMC6355031; doi:10.1371/journal.pntd.0007087)

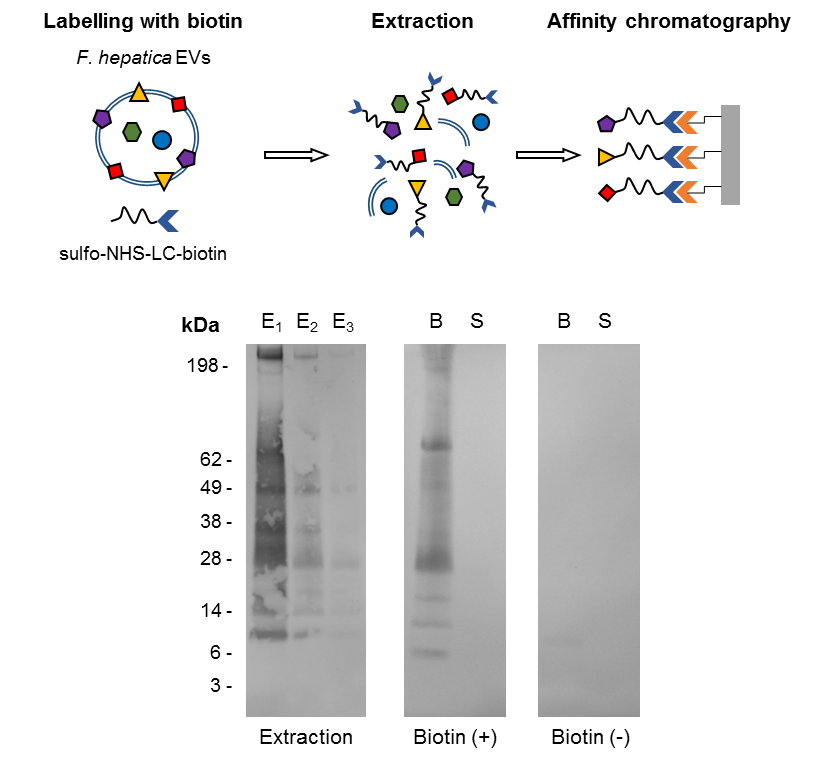

Supplement: S1 Fig — F. hepatica 15k and 120k EVs were incubated with a non-permeable biotin reagent. The EVs were solubilised with detergent and the biotinylated surface proteins recovered using a streptavidin affinity column. Western blot with streptavidin conjugated with alkaline phosphatase showing the profile of biotinylated protein after sequential extraction. E1-E3, represent each extraction step. Biotinylated proteins are purified with high efficiency after the pull-down with streptavidin agarose beads (B) whilst non-biotinylated proteins are lost in the flow-through (S). (TIF) [file pntd.0007087.s002.tif]

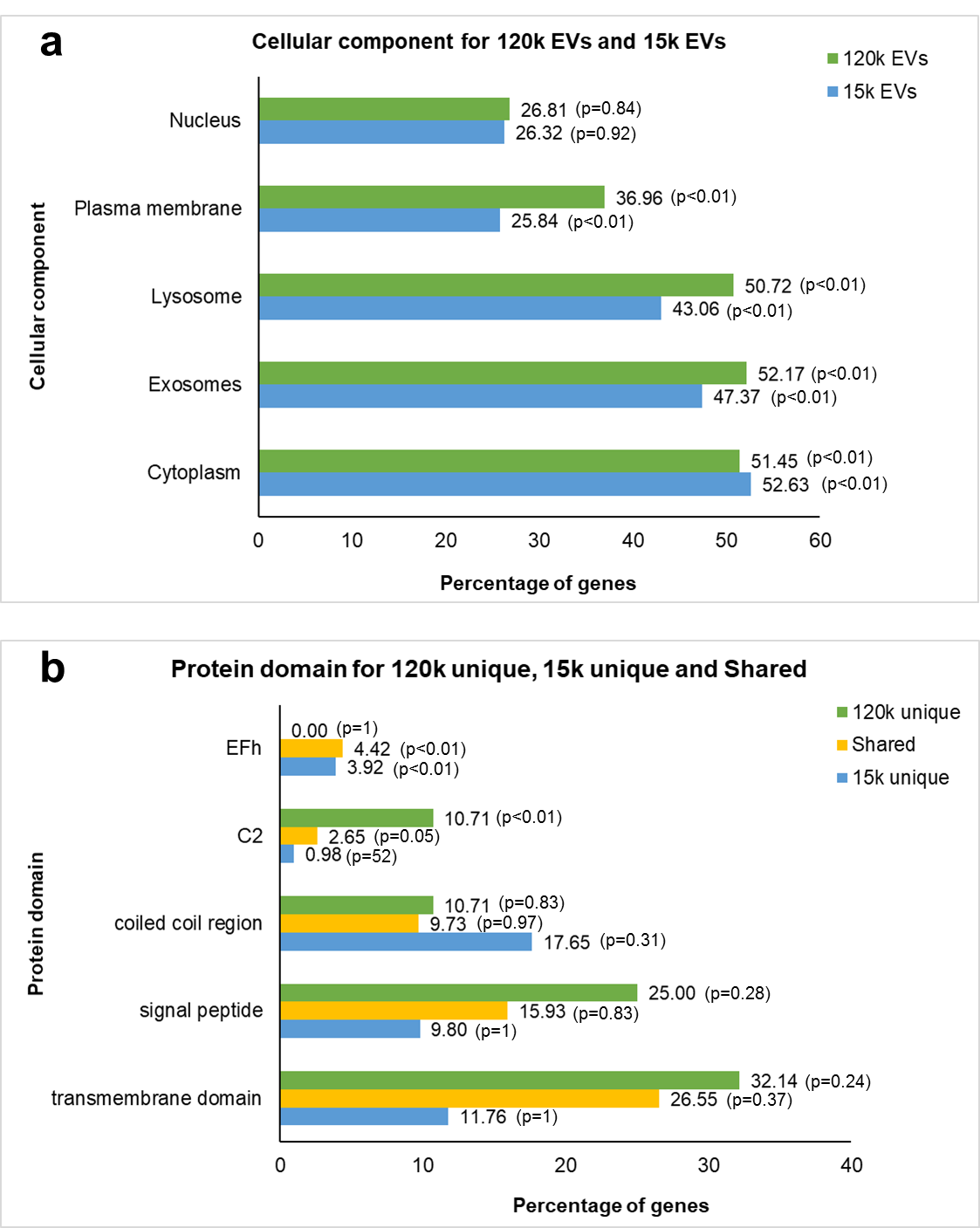

Supplement: S2 Fig — Enrichment of GO terms (A) and protein domains (B) for the proteins identified on the surface of the 15k and 120k EVs. (TIF) [file pntd.0007087.s003.tif]

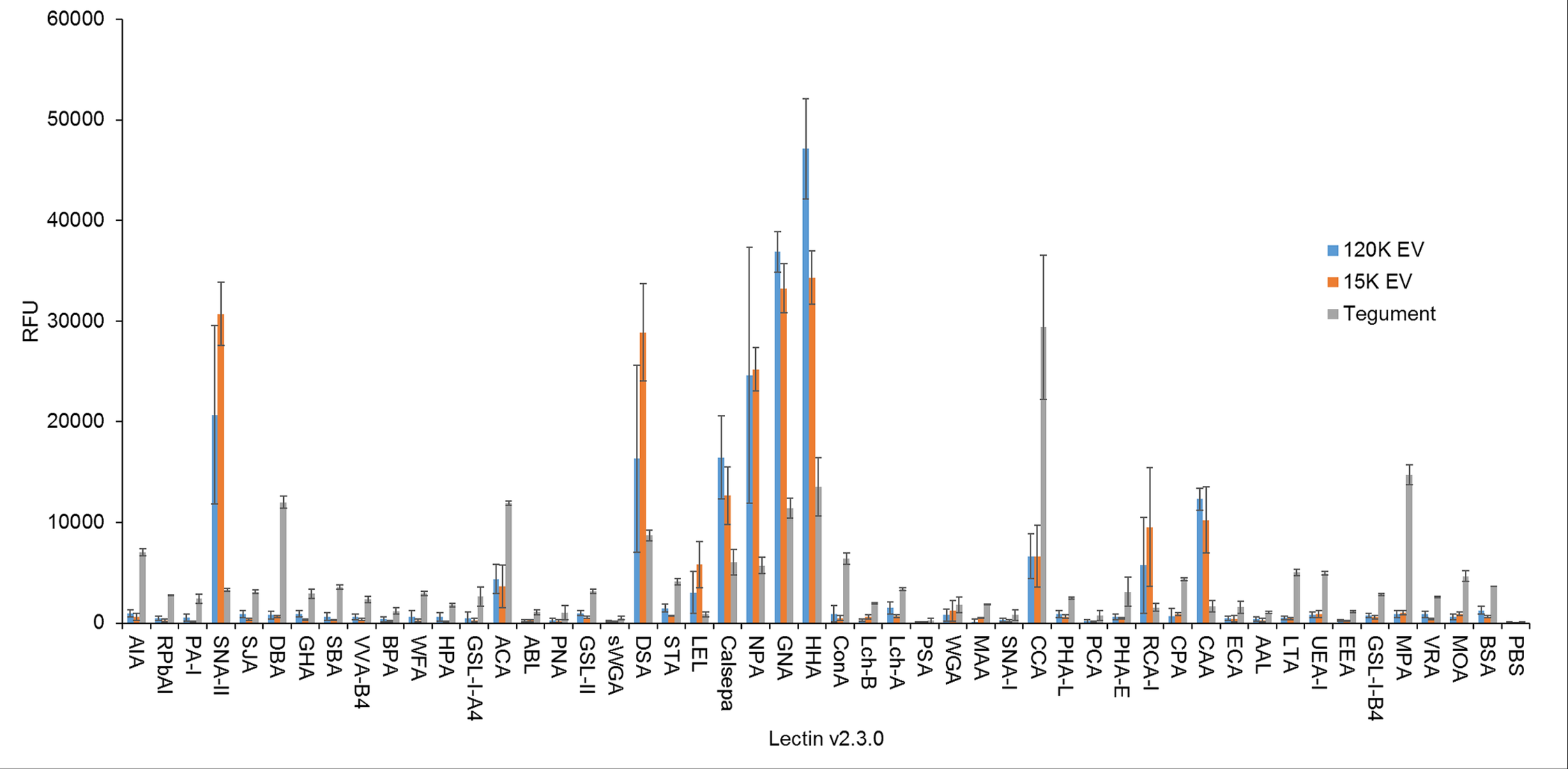

Supplement: S3 Fig — Data subjected to total intensity mean normalization. Error bars represent +/- one standard deviation. (TIF) [file pntd.0007087.s004.tif]

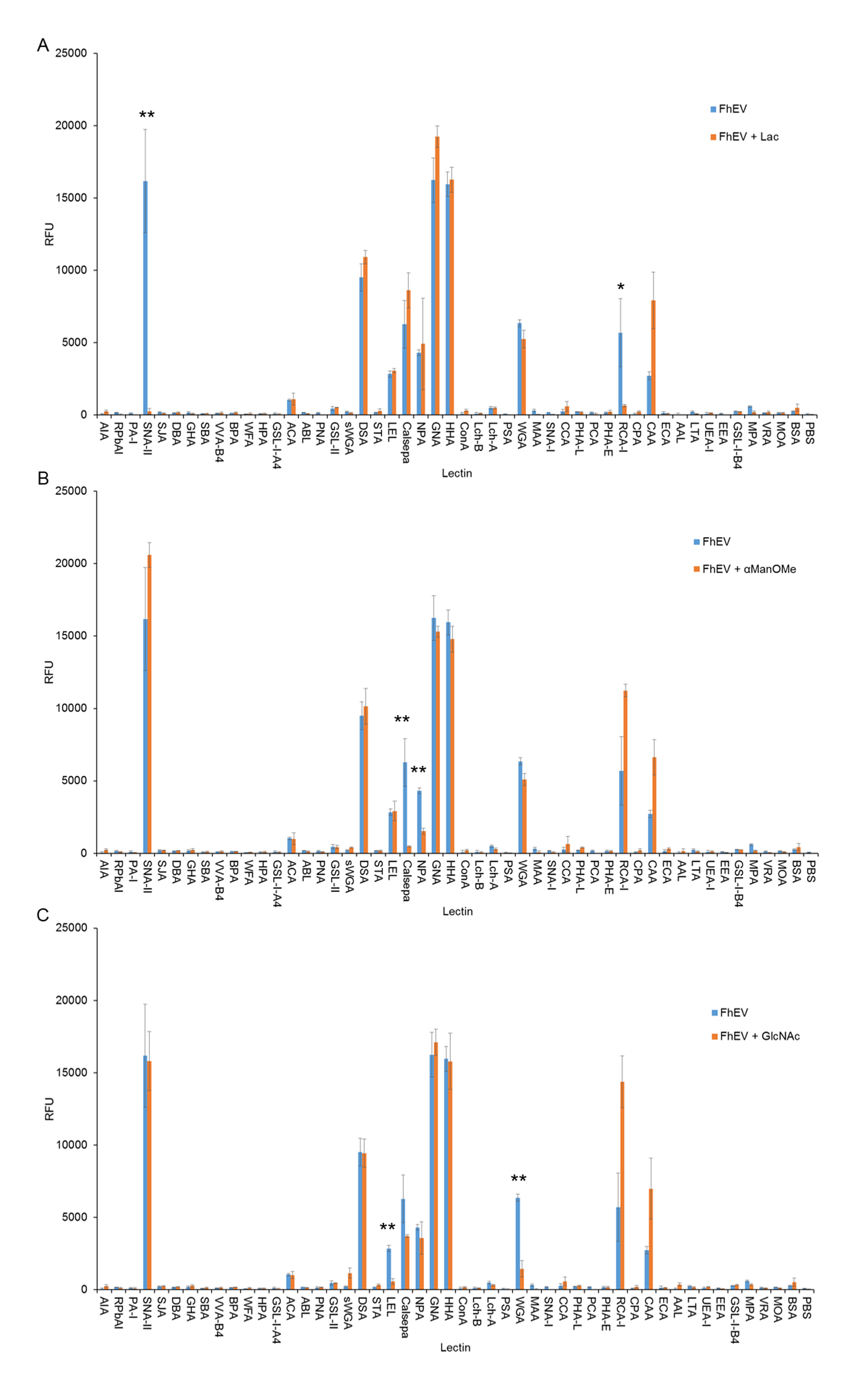

Supplement: S4 Fig — 120k EV mean lectin microarray response changes imparted by competitive inhibition with 50 mM final concentrations of A. Lac, B. αManOMe and C. GlcNAc. Error bars represent +/- one standard deviation based on three technical replicates. Significance of inhibition * p ≤ 0.05; ** p ≤ 0.01. (TIF) [file pntd.0007087.s005.tif]

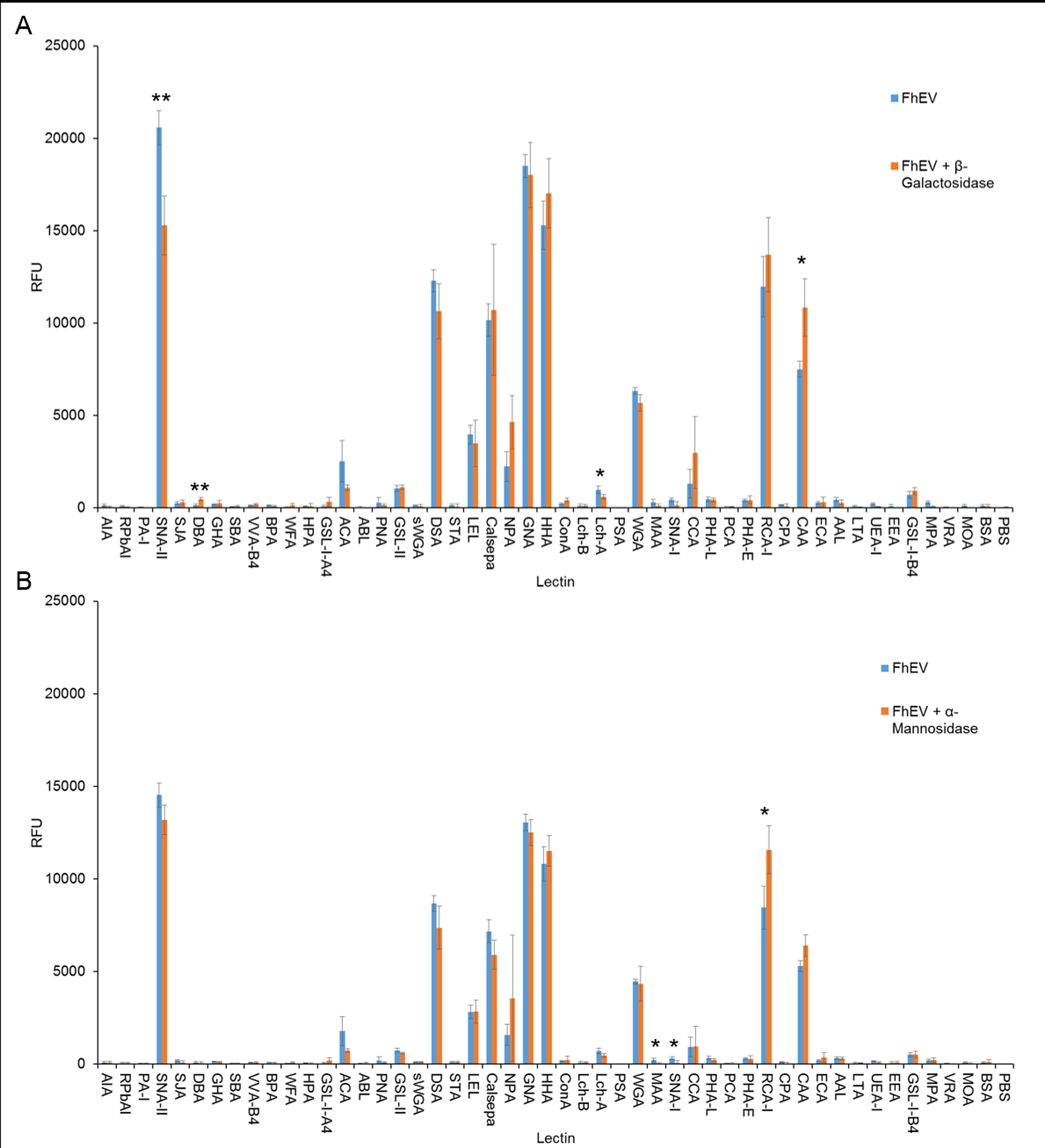

Supplement: S5 Fig — A. 120k EV mean lectin microarray responses imparted by exo-glycosidase treatment with (A) β-galactosidase (n = 5) and (B) α-mannosidase (n = 5) and their respective pH-matched controls (controls n = 3). Error bars represent one +/- standard deviation. (TIF) [file pntd.0007087.s006.tif]

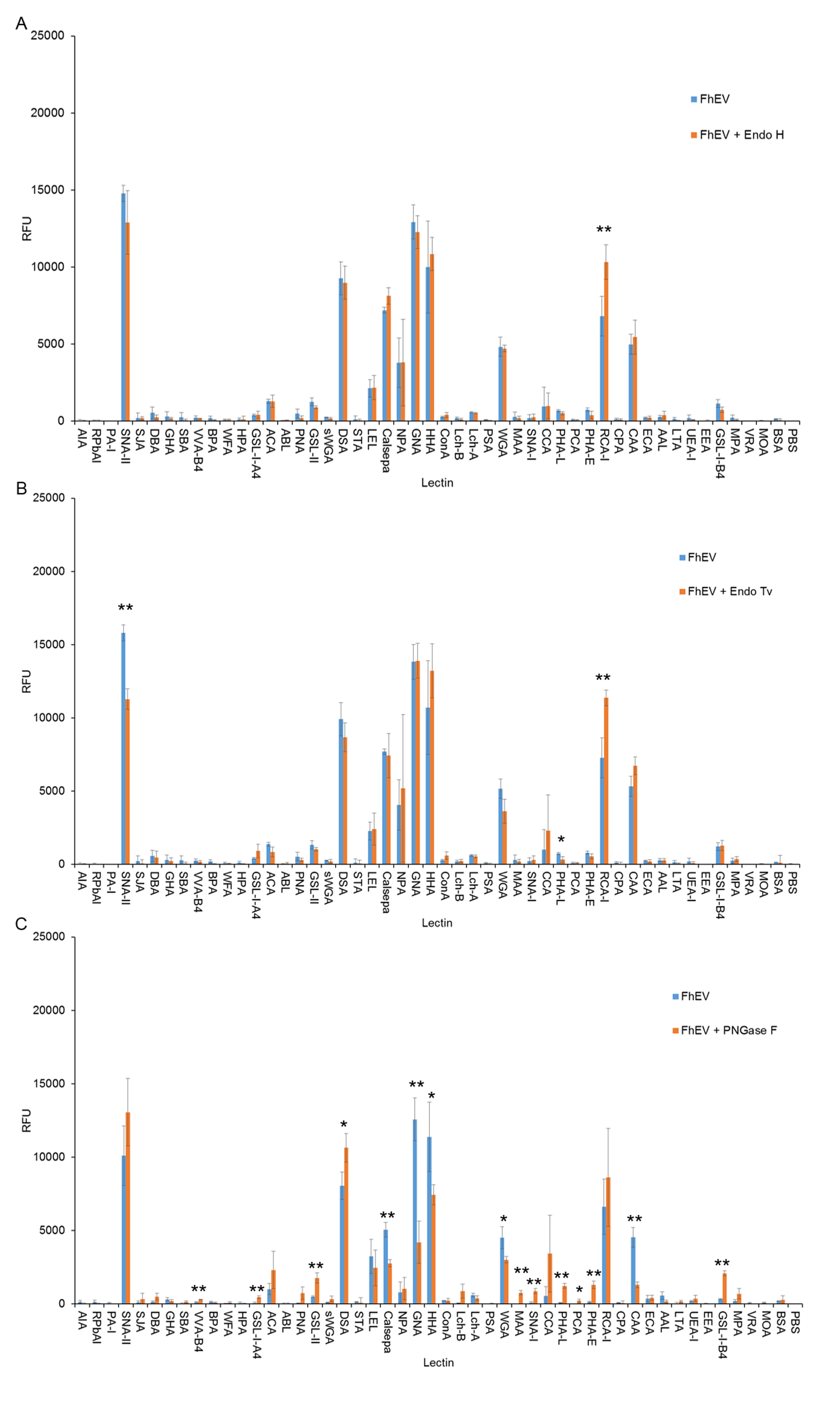

Supplement: S6 Fig — 120k EV mean lectin microarray responses imparted by treatment with endo-glycosidases A. Endo H (n = 4) and B. Endo Tv (n = 4) and glyco-amidase C. PNGase F (n = 4) and their respective pH-matched controls (all n = 3). Error bars represent +/- one standard deviation. (TIF) [file pntd.0007087.s007.tif]

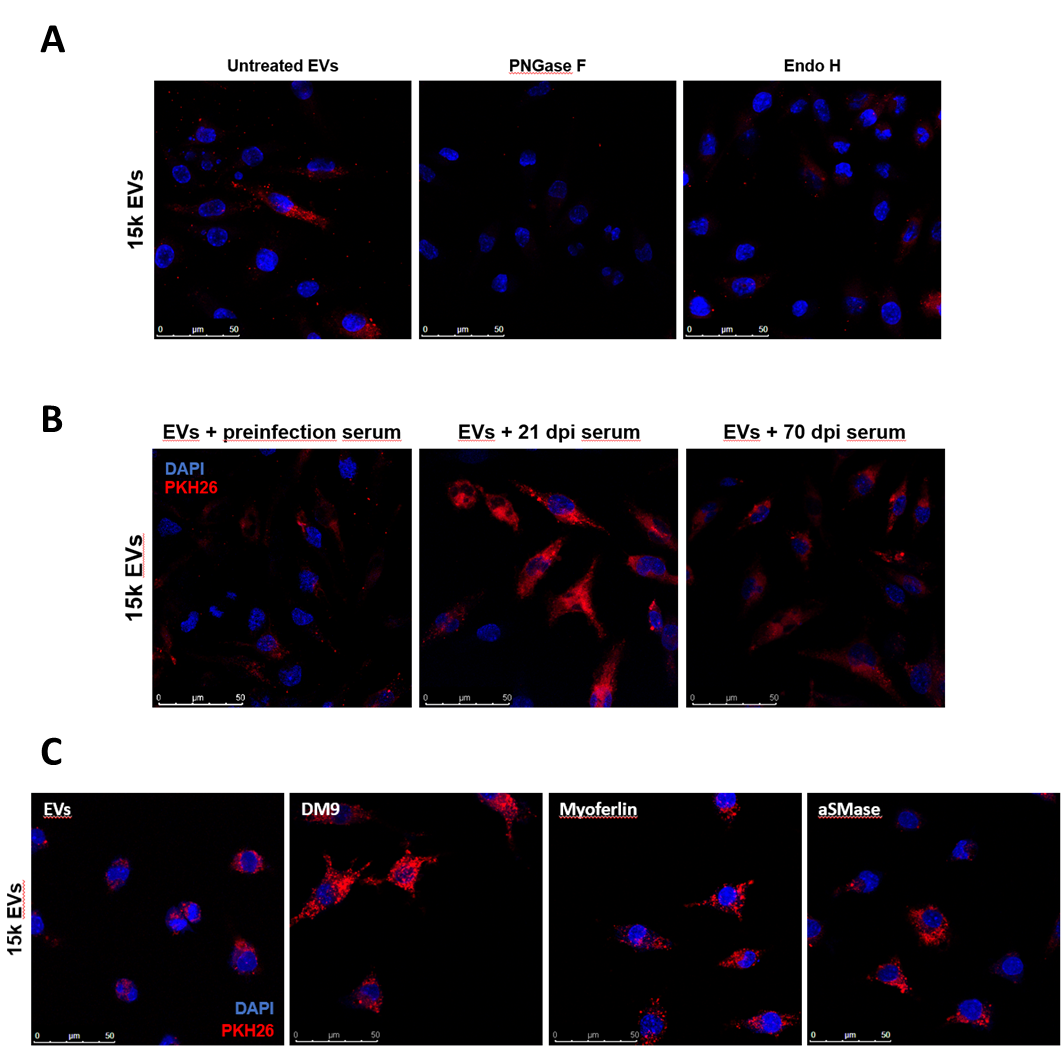

Supplement: S7 Fig — RAW264.7 macrophages were incubated with PKH26-labelled 15k EVs for 3 h at 37°C and the cells were analysed by confocal microscopy. (A) Pre-treatment of labelled EVs with the glycosidases PNGase F and Endo H considerably reduced their uptake. (B) Pre-treatment of labelled EVs with 21-day rat serum increased the internalisation of the EVs considerably. A similar, but less dramatic, effect was observed when EVs were pre-treated with the 70-day serum. (C) Pre-treatment of labelled EVs with antibodies raised against specific F. hepatica proteins (DM9, myoferlin and aSMase) resulted in a greater level of internalisation by macrophages compared to control EVs (left panel). (TIF) [file pntd.0007087.s008.tif]

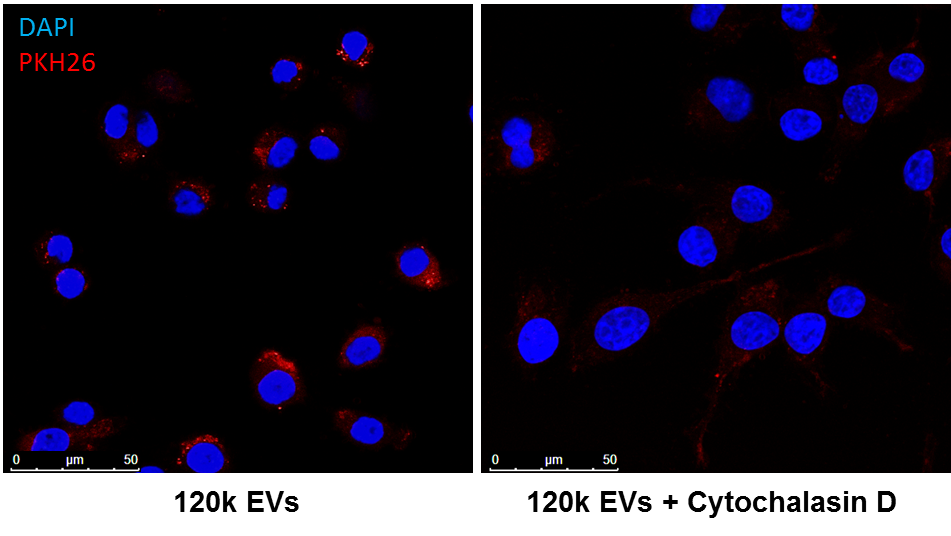

Supplement: S8 Fig — RAW264.7 macrophages were incubated with PKH26-labelled 120k EVs for 3 h at 37°C and the cells were analysed by confocal microscopy. Co-incubation of cells with cytochalasin D (2μg/ml) inhibited uptake of EVs. (TIF) [file pntd.0007087.s009.tif]

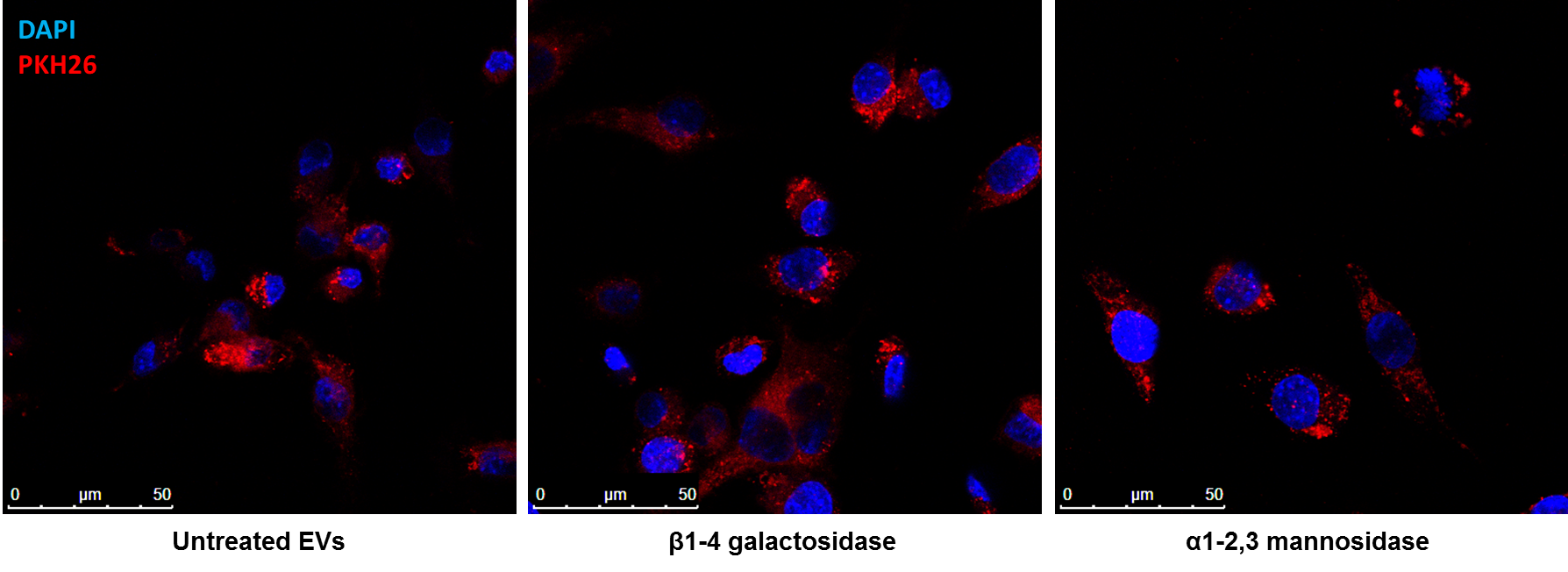

Supplement: S9 Fig — RAW264.7 macrophages were incubated with PKH26-labelled 120k EVs for 3 h at 37°C and the cells were analysed by confocal microscopy. Pre-treatment of labelled EVs with the exo-glycosidases α1–2,3 mannosidase and β1–4 galactosidase had no effect on the uptake of the EVs. (TIF) [file pntd.0007087.s010.tif]
